# Small-Molecule Inhibitors of Dengue-Virus Entry

**DOI:** 10.1371/journal.ppat.1002627

**Published:** 2012-04-05

**Authors:** Aaron G. Schmidt, Kyungae Lee, Priscilla L. Yang, Stephen C. Harrison

**Affiliations:** 1 Jack and Eileen Connors Laboratory of Structural Biology, Department of Biological Chemistry and Molecular Pharmacology, Harvard Medical School, Boston, Massachusetts, United States of America; 2 New England Regional Center of Excellence in Biodefense and Emerging Infectious Diseases (NERCE/BEID), Harvard Medical School, Boston, Massachusetts, United States of America; 3 Department of Microbiology and Molecular Genetics, Harvard Medical School, Boston, Massachusetts, United States of America; 4 Howard Hughes Medical Institute, Harvard Medical School, Boston, Massachusetts, United States of America; NIH, United States of America

## Abstract

Flavivirus envelope protein (E) mediates membrane fusion and viral entry from endosomes. A low-pH induced, dimer-to-trimer rearrangement and reconfiguration of the membrane-proximal “stem" of the E ectodomain draw together the viral and cellular membranes. We found stem-derived peptides from dengue virus (DV) bind stem-less E trimer and mimic the stem-reconfiguration step in the fusion pathway. We adapted this experiment as a high-throughput screen for small molecules that block peptide binding and thus may inhibit viral entry. A compound identified in this screen, 1662G07, and a number of its analogs reversibly inhibit DV infectivity. They do so by binding the prefusion, dimeric E on the virion surface, before adsorption to a cell. They also block viral fusion with liposomes. Structure-activity relationship studies have led to analogs with submicromolar IC_90_s against DV2, and certain analogs are active against DV serotypes 1,2, and 4. The compounds do not inhibit the closely related Kunjin virus. We propose that they bind in a previously identified, E-protein pocket, exposed on the virion surface and although this pocket is closed in the postfusion trimer, its mouth is fully accessible. Examination of the E-trimer coordinates (PDB 1OK8) shows that conformational fluctuations around the hinge could open the pocket without dissociating the trimer or otherwise generating molecular collisions. We propose that compounds such as 1662G07 trap the sE trimer in a “pocket-open" state, which has lost affinity for the stem peptide and cannot support the final “zipping up" of the stem.

## Author Summary

Fusion of viral and cellular membranes is necessary to establish infection by an enveloped virus. This process is facilitated by rearrangement of protein(s) present on the virion surface in response to molecular cues from the compartment from which fusion occurs, such as low pH of an endosome. Dengue virus is an enveloped virus in the flavivirus family; its “E" (for envelope) protein is the fusion mediator. We previously showed that peptides derived from the membrane proximal “stem" of the E protein bind a form of E that represents a late-stage fusion intermediate. We used this assay to screen for small-molecule inhibitors that compete for stem-peptide association with E. We describe one such inhibitor and its analogs that block viral fusion. These inhibitors also block infectivity if added to dengue virus before infection. Withdrawing the inhibitor before fusion reverses the blockage. We propose that these small molecules bind a hydrophobic pocket on the virion surface and that the virus carries them into the endosome, where they prevent viral fusion by stabilizing an intermediate conformation of the E protein that cannot complete the fusion-promoting conformational change. Identification of these fusion inhibitors shows that viral entry is a possible target for anti-flavivirus drugs.

## Introduction

Enveloped viruses penetrate into the cytosol of their target cell by fusion of viral and cellular membranes [1], [2]. Flaviviruses, such as dengue, penetrate from endosomes, following uptake by clathrin-mediated endocytosis [3], [4]. At endosomal pH, proton binding by their envelope protein, E, triggers a fusion-promoting conformation change [5], [6].

The flavivirus envelope fusion protein, E, forms a well-ordered lattice of 90 dimers on the surface of a mature, infectious virus particle [2], [7]. Crystal structures of soluble forms of E (“sE"), which include the first ∼395 of ∼445 ectodomain residues but lack a conserved, membrane-proximal “stem" region, have contributed to molecular descriptions of flavivirus fusion [8]–[12]. The three domains (DI–III) of the E protein reorient with respect to one other during the fusion-promoting conformational transition, which includes dissociation of the prefusion dimer and reconfiguration of the subunits into trimers [2]. At an intermediate stage a hydrophobic “fusion loop" at one end of the extended E subunit inserts into the outer leaflet of the target bilayer [2], [13]. The driving force for pinching the two membranes together appears to come from contacts made by domain III, as it folds back against domain I, and by the stem, as it “zips" up along adjacent domain II monomers [1], [2].

Molecular understanding of the fusion pathway and the proteins involved has enabled discovery of small-molecule and peptide inhibitors that target intermediates in these fusion-inducing rearrangements. The best-known example of the latter type of entry inhibitor is T-20/enfuvirtide, a peptide used to treat HIV-1 infection [14]–[17]. The T-20 peptide interferes with a late stage in the fusion-inducing conformational transition of HIV-1 gp41. Certain small molecules block HIV-1 fusion by a similar mechanism, binding in a conserved pocket on the gp41 inner core [18]. Inhibitors that target the fusion glycoprotein, F1, of respiratory syncytial virus (RSV) also prevent infection by blocking a conformational transition [19], [20].

Targeting the HIV-1 and RSV glycoproteins is possible, because fusion occurs at the plasma membrane, where exposure of the relevant fusion intermediates allows straightforward access to the specific inhibitors. For viruses such as flaviviruses that fuse from endosomal compartments, however, targeting an intermediate of the rearranging fusion protein requires concentrating the inhibitor within the endosome, as its potential binding sites are not available until reduced pH has induced their exposure.

Kielian and co-workers have reported reconstitution of an sE trimer for both alpha- and flavivirus envelopes, suggesting that one might use reconstitution strategies to identify inhibitors that block steps in fusion [21], [22]. We found recently that we could target a fusion intermediate of dengue virus E with peptides derived from its ectodomain stem [23], [24]. These peptides bind the postfusion form of DV2 sE trimer, mimicking late steps in stem rearrangement. They inhibit *in vitro* fusion and DV2 infectivity. C-terminal modification with membrane targeting sequences increases their inhibitory strength [23]. A series of experiments support a two-step mechanism, in which a reversible, non-specific interaction with the viral membrane brings virion-associated peptides into the low-pH endosome, where full exposure of the peptide site on the E-protein conformational intermediate leads to tight, specific binding, which interfere with the final “zipping" of the stem [24].

Can small molecules also inhibit this step in the fusion pathway? We have adapted the assay we used to study interaction of stem-derived peptides with stem-less sE trimer, to screen for small-molecule inhibitors that target this fusion intermediate. We have identified compounds that compete for stem peptide association, and we show that they reversibly inhibit DV infectivity. We further show, using model liposomes, that these molecules specifically block viral fusion. They appear to bind the virion before adsorption to cells, by interacting with the prefusion, dimeric E protein, possibly in a previously identified, hydrophobic pocket. This association presumably permits their virus-associated transfer into endosomes. Limited structure-activity relationship studies have yielded compounds that inhibit DV2 (NGC isolate) with IC_90_∼1 µM. Our competition screen has thus identified a group of potent, small-molecule inhibitors of DV entry validating experimental screens for small molecules that block viral entry from internal compartments.

## Results

### High-throughput screen (HTS) for DV fusion inhibitors

**Figure 1:**
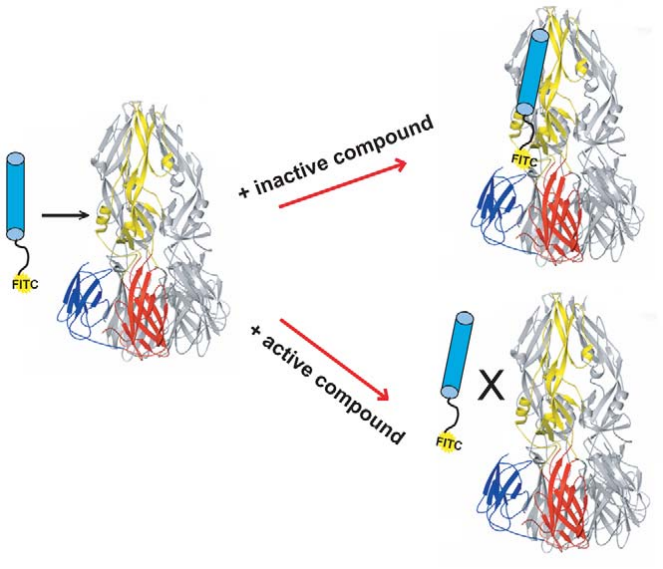
Schematic of high-throughput screening platform. DV2 sE_3_ with one monomer colored by domain: DI (red), DII (yellow) and DIII (blue). Stem peptide DV2^419–447^, with FITC at its N-terminus, is represented as a cylinder, conjugated at its N-terminus with FITC. Addition of small molecules from screening libraries either affect (active compounds) or do not affect (inactive compounds) interaction of stem peptide with sE_3_.

**Figure 2:**
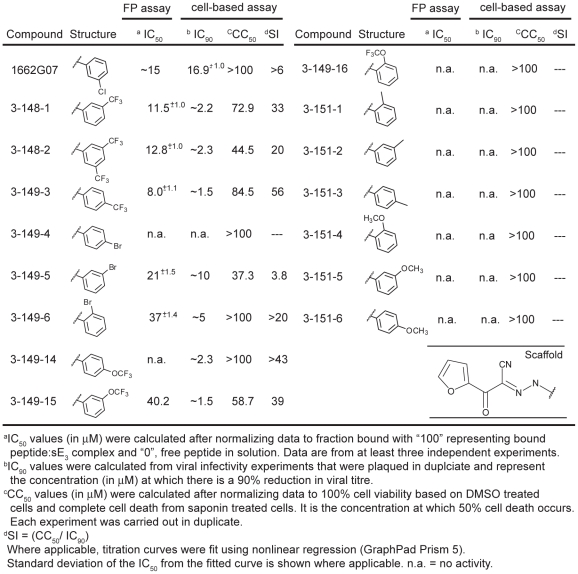
Biochemical, cytotoxicity and antiviral summary of 1662G07 select compounds from the 3-148, 149 and 151 series.

We have described a fluorescence polarization (FP) assay to identify stem-derived peptides that bind the trimeric postfusion conformer of DV2 sE [24]. We found peptides from the C-terminal stem region that bind tightly to this proposed fusion intermediate. With one such peptide, DV2^419–447^, tagged at its N-terminus with FITC, we adapted the FP assay to screen for small molecules that compete for binding to trimeric sE (Figure 1). We screened ∼30,000 compounds in a 384-well format and found several that were active, as measured by a reduction in the fluorescence polarization signal. We chose to pursue further work on one such “hit", 1662G07 (Figure 2), for which several structurally similar compounds were commercially available.

We tested a set of related compounds, both from the screening libraries and from commercial vendors, to obtain preliminary structure activity relationships (SAR). Several modifications to the parental scaffold affected competition with peptide (Table S1). Modification or removal of the nitrile moiety (R1 position) impaired or abolished activity. Removal of the halogen in the *m*-position on the R2 (as phenyl) decreased competition; addition of strongly electron withdrawing groups (either trifluoro- or trifluoromethoxy) in the *o*- or *m*-positions increased it. A thiophene at R3 was equivalent to the furan in 1662G07, but a cyclopropane in that position eliminated competition with peptide.

### Directed synthesis of 1662G07 analogs

From preliminary SAR analysis with commercial analogs described above, we chose to synthesize two series of compounds based on the parental scaffold. Series 3-148, 3-149 and 3-151 varied the R2 position while the 3-110 varied at the R3 positions. (Figures 2 and 4, Tables S2 and S3). Among the 51 compounds from these series, we found that sixteen competed with stem-derived peptide for binding to the DV2 sE trimer. Strongly electron withdrawing groups in the *o-*, *m-*, or *p-* positions on R2 (as phenyl) enhanced activity; substitution with methyl or methoxy-methyl groups at these positions yielded inactive compounds. Some larger, heterocyclic rings impaired activity (Figures 2 and 4. Tables S2 and S3).

### 1662G07 analogs inhibit DV2 infectivity

**Figure 3:**
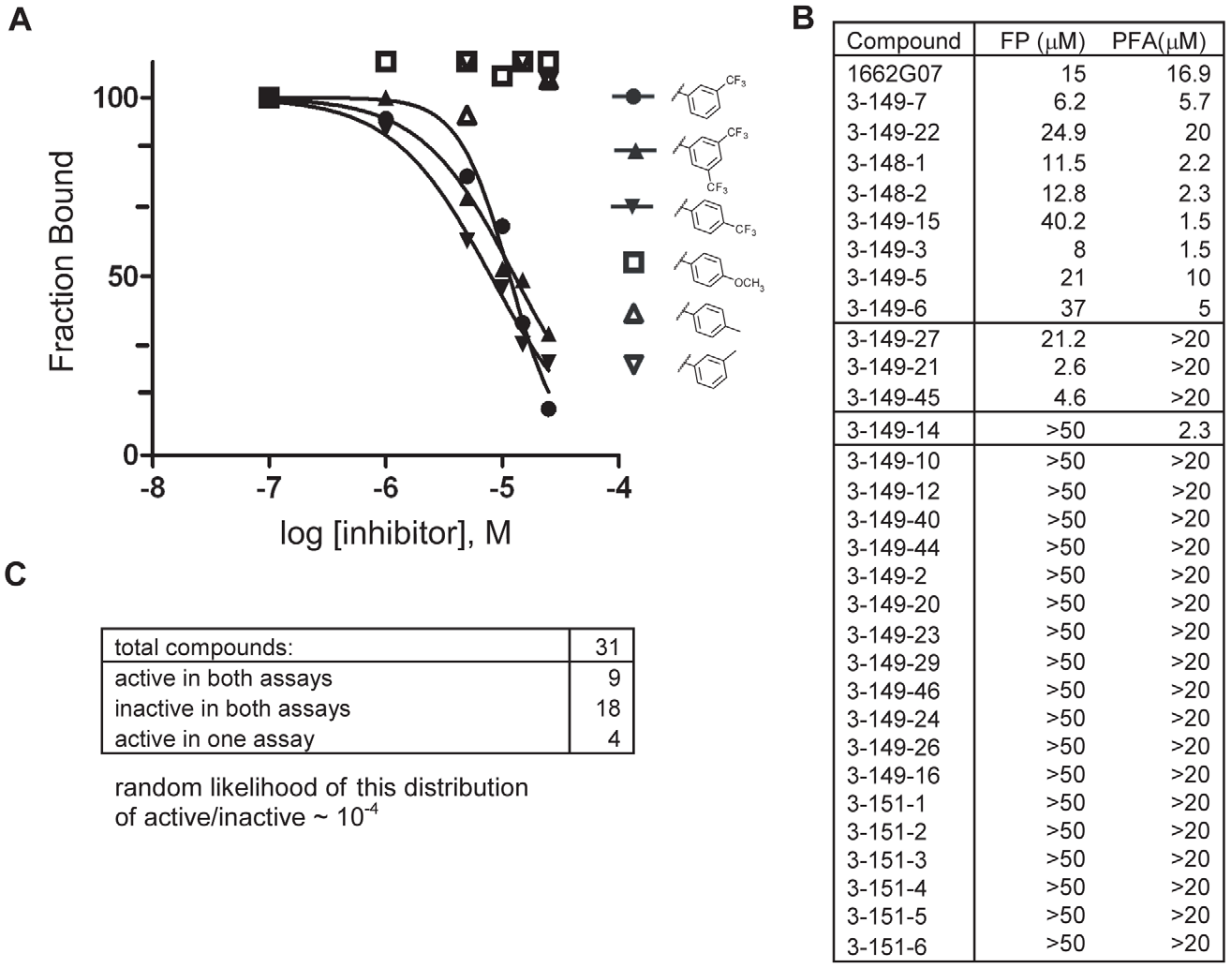
Activity profiles of selected compounds in competition FP and PFA assays. (A) Representative FP titration curves of active analogs: 3-148-1, 3-148-2 and 3-149-3 and inactive analogs: 3-151-2, 3-151-3 and 3-151-6 and their competition with the interaction between stem peptide and sE_3_. (B) Activities in the two assays for all compounds in the SAR series which varied at R2 (as phenyl). Table 1 and Supplementary Table 2 give the chemical structures of all compounds in this list. (C) Summary of the numbers of active and inactive compounds in each of the two assays. The likelihood that this degree of concordance could result from random pairing of unrelated activities is less than 10^−4^. Only 9 of the 31 compounds had activities within the range of achievable concentrations, making a quantitative correlation of those activities uninformative, particularly in few of other sources of error such as nonspecific binding to cell surfaces in the case of the infectivity assay and limited solubility of some compounds in the case of both assays.

**Figure 4:**
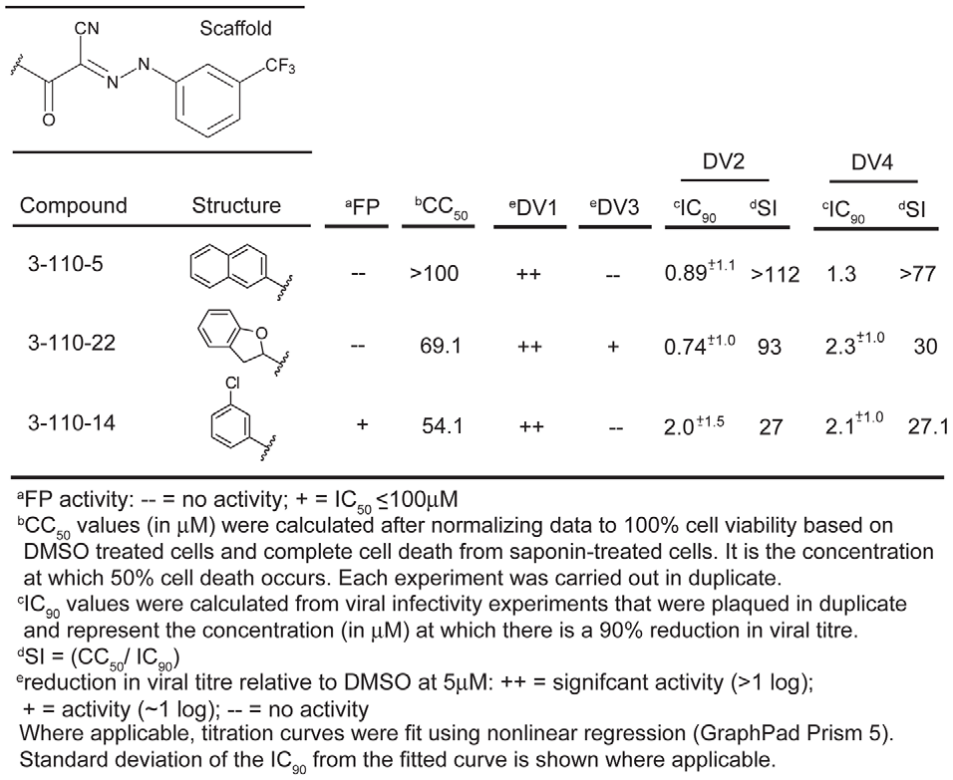
Biochemical, cytotoxicity and antiviral summary of selected compounds from the 3-110 series.

Do these small molecules, identified in a screen for binding to a late-stage fusion intermediate, inhibit DV2 viral infectivity? We used a standard plaque forming assay to test the effect of the analogs from the 3-148, 3-149, 3-151 and 3-110 series at a single concentration on growth of DV2 NGC. The virus inoculum was preincubated with compound for 15 minutes and then adsorbed to BHK-21 cells, at a multiplicity of infection (MOI) of 1, for 1 hour at 37°C. Supernatants were harvested after 24 hours and titred by standard plaque assay [23]. A subset of the compounds from both series reduced DV2 infectivity (Figures 2 and 4, Tables S2 and S3). Compounds were inactive against vesicular stomatitis virus, an unrelated enveloped virus and were noncytotoxic at the concentrations tested (Figure S7, Figures 2 and 4, Tables S1, S2 and S3). Comparison of the activity profiles from the 3-148, 3-149 and 3-151 series in the peptide-competition and viral infectivity assays revealed a clear concordance between active compounds in one assay and actives compounds in the other (Figure 3). The likelihood that this degree of concordance could be random is less than 10^−4^.

Compounds in the 3-110 series were particularly active against DV2. We therefore tested this series at a single concentration (5 µM) against isolates from the other three dengue serotypes: DV1 WP74, DV3 THD 3 and DV4 TVP360. The compounds of series 3-110 inhibit DV1, 3 and 4 infectivity to varying degrees (Figures 4 and S1 and Table S3). DV3 was particularly insensitive to inhibition, as we had also found when testing its response to stem-derived peptides. From this initial screen, we looked more closely at three analogs that appeared to have the strongest antiviral effect, 3-110-5, 3-110-14 and 3-110-22. We examined their inhibition of DV2 and DV4 viruses to determine IC_90_s. As seen in Figure 4 these compounds had strong antiviral activity against DV2 and DV4, with IC_90_s in submicromolar and micromolar ranges, respectively. At the same concentration used with the DV serotypes, none of the compounds had detectable activity against Kunjin, a subtype of West Nile virus (Figure S2); the analogs most potent for inhibiting dengue, 3-110-5, 3-110-14 and 3-110-22, had no effect on Kunjin, even at 20 µM.

**Figure 5:**
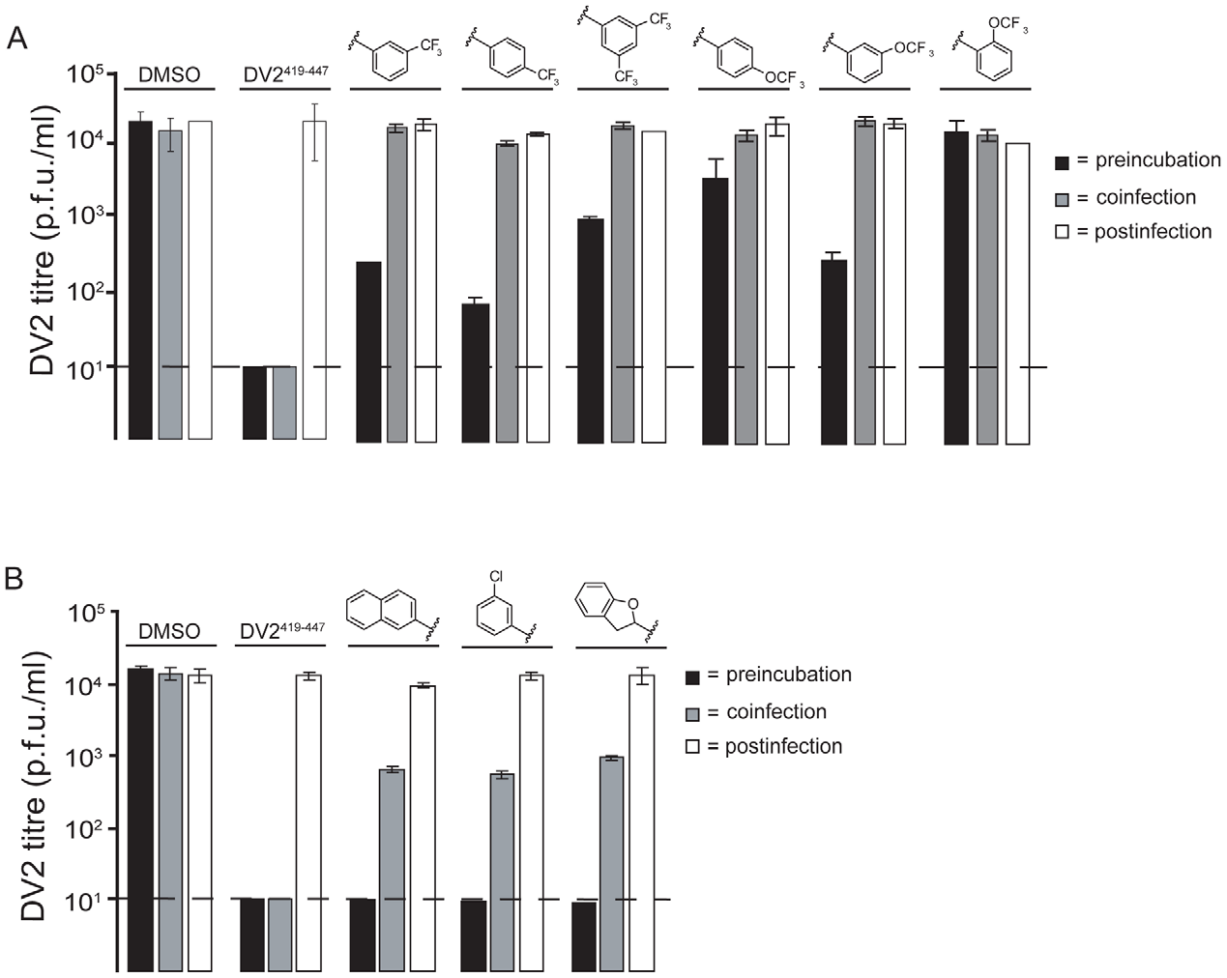
Effect of order-of-addition on small-molecule inhibition. (A) Comparison of *o*-, *m*-, and *p*-OCF_3_ and *m*-, di-*m*- and *p*-CF_3_ substitution from the 3-148 and 3-149 series (B) Comparison of compounds from the 3-110 series. Preincubation: addition of 1662G07 analogs to inoculum 15′ before adsorption to cells. Coinfection: addition of analogs at the time of adsorption. Postinfection: addition of analogs one hour after adsorption of virus. In all cases, cells were washed with PBS before adding compounds. Supernatants were harvested after 24 hours and viral titres determined by standard plaque forming assay (done in duplicate). Compounds from (A) and (B) were used at 15 and 5 µM, respectively. DV2^419–447^ stem peptide at 1 µM was used as a control.

The small-molecule inhibitors were selected in a screen that detects formation of an E-protein conformation adopted only after a virion has arrived in the low-pH environment of an endosome – an intracellular compartment presumably inaccessible to the free compounds. A series of order-of-addition experiments using the 3-148, 3-149 and 3-110 series show that to have a significant inhibitory effect, the compounds must be preincubated at 37°C with the viral inoculum before adsorption to cells (Figure 5). We observed the same level of inhibition using a direct plaque assay as a readout (Figure S8). When compound and virus inoculum were added to cells at the same time, we detected an approximately tenfold drop in viral titre compared with the DMSO control for compounds in the 3-110 series and little or no effect for compounds in the 3-148 and 3-149 series. Postinfection treatment of cells with compound one hour after initial adsorption of virus did not reduce viral titre, nor did pretreatment of cells for one hour before virus adsorption (Figure 5 and data not shown). These results imply a direct association of compound and virion before endocytosis of the virus.

### Inhibition of fusion

**Figure 6:**
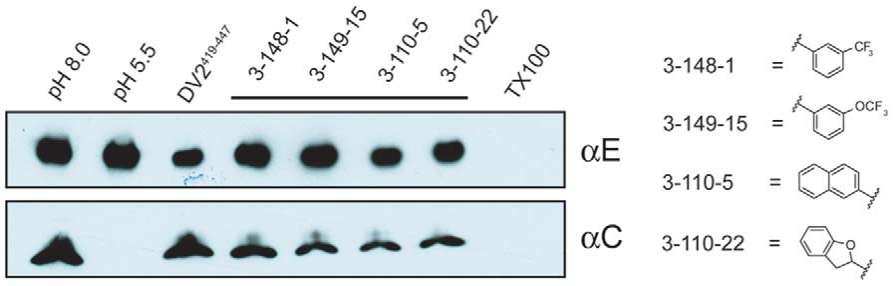
Inhibition of viral fusion with liposomes. Effect on content mixing of preincubating virus with 1662G07 analogs. Virus and analogs 3-148-1, 3-149-15, 3-110-5 and 3-110-22 (all at 50 µM) were incubated with liposomes encapsulating trypsin and acidified to pH=5.5. Following back-neutralization and incubation for 1 hr at 37 C, samples were prepared for SDS-PAGE and immunoblotted with αC and αE antibody. Fusion leads to exposure of core protein to trypsin and loss of the corresponding band but retention of the envelope protein band. DV2^419–447^ stem peptide, at 1 µM, was used as a positive control.

To detect DV2 fusion with liposomes, we used the content-mixing assay we previously applied to characterize peptide inhibitors of DV [24]. Selected compounds from the 3-148, 3-149 and 3-110 series were incubated for 15 minutes at 37°C with virus, which was then added to trypsin-loaded liposomes. We adjusted the pH of the medium to ∼5.5 for 10 minutes, back-neutralized the samples, and incubated for an additional 45 minutes at 37°C to allow trypsin to act. Digestion of the viral core protein, which would have been exposed to protease only after fusion of virions with liposomes, was assessed by SDS-PAGE and immunoblotting. Protection of the core protein from proteolysis with retention of the envelope protein indicated an effective fusion inhibitor. A subset of the compounds we tested specifically blocked content mixing of virus with trypsin-loaded liposomes (Figure 6). We used stem peptide DV2^419–447^, previously shown to inhibit fusion, as a positive control.

### Interaction of inhibitory compounds with a DI/DII fragment of E

**Figure 7:**
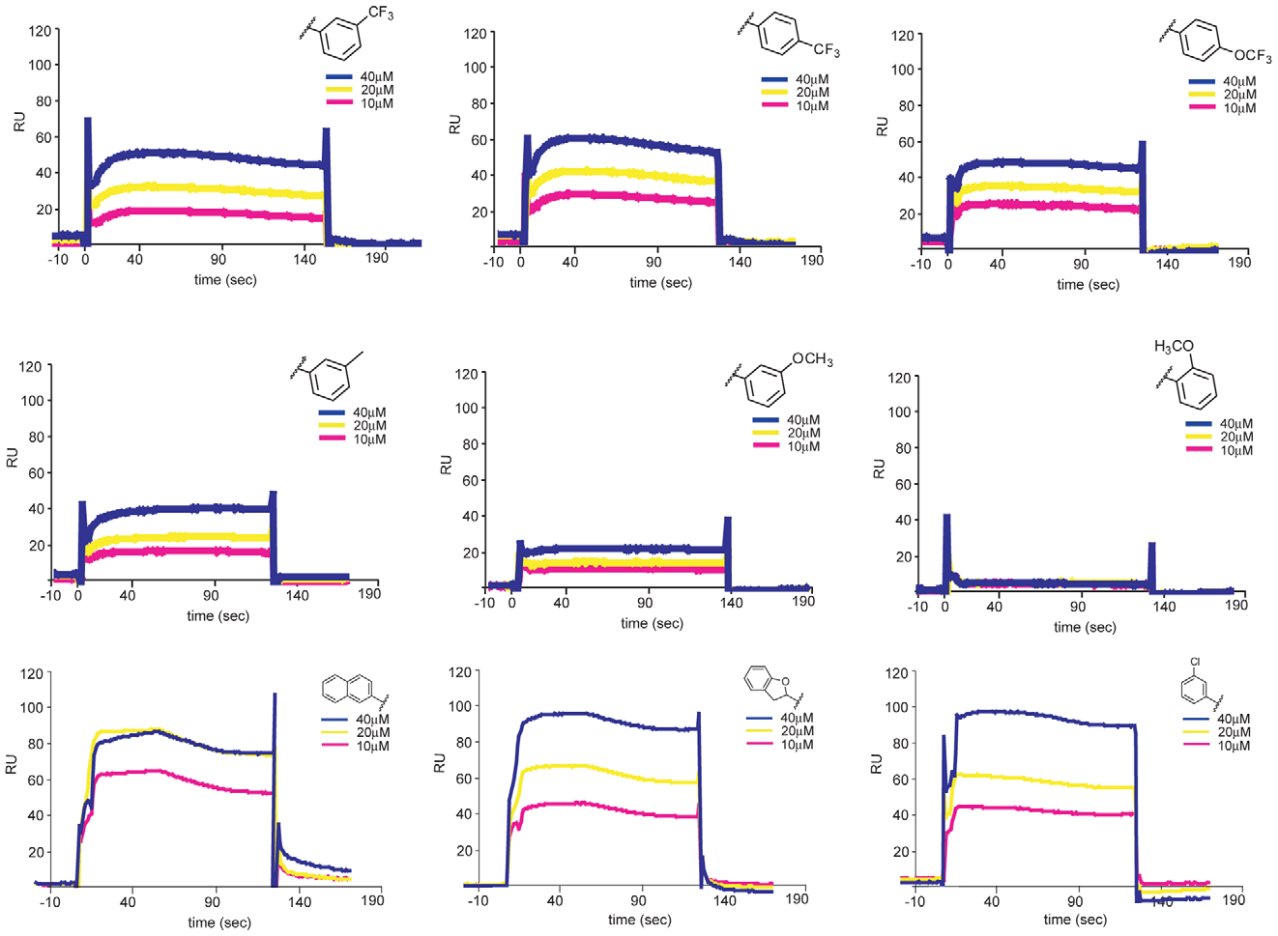
Interaction of 1662G07 analogs with DI/DII. DI/DII was immobilized on a CM5 sensorchip. Analogs 3-148-1, 3-149-3, 3-149-14, 3-151-2, 3-151-2, 3-151-5, 3-151-4, 3-110-5, 3-110-14 and 3-110-22 were passed over the DI/DII surface at 10, 20 and 40 µM. Background for nonspecific binding to the chip surface was corrected for by passing the analogs over a protein-free channel. All measurements carried out in duplicate.

The assay we used to find inhibitory compounds detects an interaction with trimeric E, which forms on virions only after exposure to low pH. Yet the inhibitory small molecules appear to bind virions at pH 7. Thus, the inhibitors can associate with both the pre- and postfusion E-protein conformers. Unless their binding site is at an interface between adjacent subunits in one of the two conformational states, we expect the compounds also to bind a monomeric form of E. We expressed and purified DI/DII, a soluble, monomeric fragment of E comprising only the first two domains (Figure S3) and showed by surface plasmon resonance (SPR) that the inhibitory small molecules from both series indeed bind directly and reversibly to DI/DII (Figure 7). Control compounds, which do not inhibit viral infectivity and do not compete for peptide binding to trimeric sE, do not bind DI/DII.

### Reversibility of binding to virions

**Figure 8:**
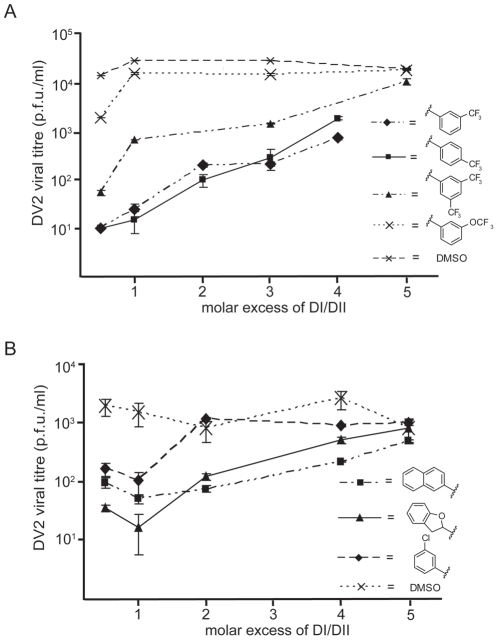
Reversibility of antiviral effect. Viral inocula were preincubated with 1662G07 analogs from the (A) 3-148 and 3-149 and (B) 3-110 series for 10′ at 37°C. DI/DII was then added in molar excess and the incubation continued for an additional 15′. Each inoculum was added to cells, and supernatants were harvested 24 hrs later. An inoculum preincubated with DI/DII alone at the same molar excess showed no loss in viral titre.

To rule out the possibility that the small molecules inactivate virions nonspecifically, we tested whether addition of DV2 DI/DII to an inoculum preincubated with a small-molecule inhibitor could restore infectivity. We incubated a virus inoculum for 10 minutes with selected compounds from the 3-148, 3-139 and 3-110 series at inhibitory concentrations and then added DI/DII in molar excess. Exogenous DI/DII indeed reversed the small-molecule inhibition (Figure 8). DV2 DI/DII alone did not affect viral titre. WNV DI/DII did not restore infectivity in the presence of these compounds (Figure S9), consistent with their failure to inhibit Kunjin virus.

## Discussion

We have shown that 1662G07 and it analogs inhibit growth of DV2 and that they block low-pH triggered fusion of virus with liposomes. We identified the parent compound, 1662G07, in a high-throughput screen that detected competition by active molecules with binding to trimeric sE of a fluorescein-tagged, stem-derived peptide. We designed this assay to represent the final step(s) in the low-pH triggered, E-protein conformational change – the process that induces penetration from endosomes through fusion of viral and endosomal membranes. Nonetheless, the inhibitory activity of these small molecules depends on binding to a virion before the virus encounters a cell, indicating that the compounds can also associate with E in its dimeric, pre-fusion conformation. Indeed, they bind in solution to a monomeric, DI/DII fragment of E, and addition of this fragment to an inoculum preincubated with one of the compounds restores infectivity, presumably by sequestering the inhibitor.

How can small molecules that bind the prefusion E dimer and the DI/DII fragment also block association of a stem-derived peptide with the sE trimer? From the known structures of sE dimers (prefusion) [9] and trimers (postfusion) [25] and from accurate docking of the former into subnanometer-resolution cryoEM reconstructions of virions [26], we can propose both an answer to this question and a model for the mechanism of action of the small-molecule inhibitors we have studied. The activity of these molecules in an assay for infectivity correlates well with their capacity to compete with a stem-derived peptide for binding to sE trimer. The most straightforward explanation for this correlation is that the compounds bind at a site accessible on both prefusion and postfusion E conformers. The most obvious site on E for small-molecule binding is a pocket, adjacent to the hinge between domains I and II, which accepts a β-octyl-glucoside (β-OG) molecule when sE dimers are crystallized in the presence of the detergent. This pocket closes down in the trimer conformation seen in the crystal structure, and the closed pocket is incompatible with occupancy by a bulky ligand (e.g., 1662G07). A dimer-to-trimer conformational transition will then require expulsion of the ligand, imposing a barrier to completion of the fusion process. For this reason, several groups have used *in silico* screens to find potential pocket-binding compounds, and in at least two cases, the results of those screens have yielded active inhibitors [27]–[31]. It has not yet been shown, however, whether the compounds found in this way indeed bind in the pocket as predicted. One of those computational screens used the Maybridge library for its search, and one of two active inhibitors identified is related to 1662G07, including most of the scaffold in Figure 2 [27]. That compound was not represented, however, in the version of the Maybridge library we used in our experimental screen. We have docked several of our compounds, using the GLIDE program [32]. We obtain fits consistent with the crystallographically observed interactions of β-OG (Figure S6).

**Figure 9:**
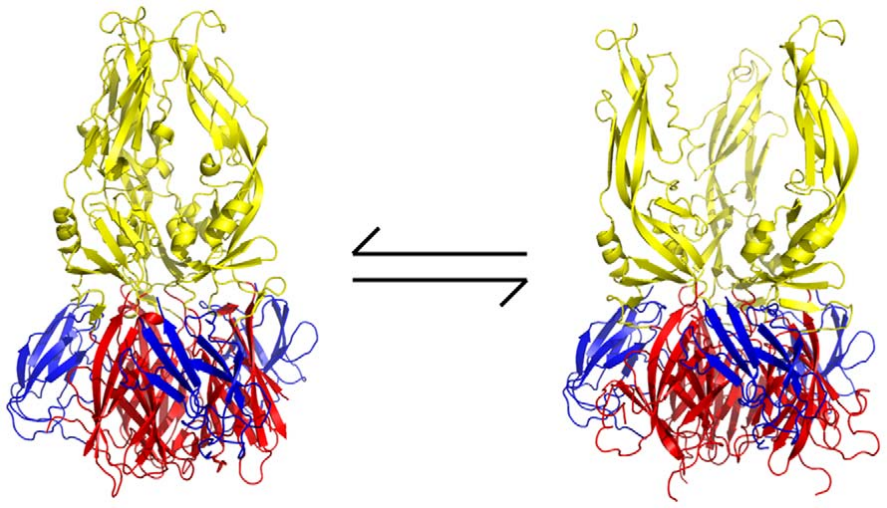
Proposed mechanism of action of small-molecule inhibitors and postulated equilibrium between two conformations of the sE trimer. In the “pocket-open", inhibitor-stabilized conformation (right image), the stem-binding groove is absent and the final fusion-inducing step in the conformational change cannot occur. Moreover, sE in this pocket-open conformation would not bind stem-derived peptides. Domains I, II and III are in red, yellow and blue, respectively. All images created with PyMol.

Although the β-OG pocket is closed in the trimer, its mouth is fully accessible. Examination of the E-trimer coordinates (PDB 1OK8) shows that conformational fluctuations around the hinge could open the pocket without dissociating the trimer or otherwise generating molecular collisions (Figure 9). We suggest that compounds such as 1662G07 inhibit peptide binding by trapping the sE trimer in a “pocket-open" state, which has lost affinity for the stem peptide and cannot support the final “zipping up" of the stem (Figure 9). Binding of a compound in the β-OG pocket can explain how it accompanies virions into endosomes. Then, even if structural rearrangements of the E protein as it transitions at low pH from dimer to trimer expel the compound from the pocket, it would still remain at relatively high concentration in the endosomal space and be able to rebind rapidly. Effective inhibition would simply require that the rate of rebinding be higher than the rate at which the stem zips up along domain II.

One potent inhibitor of infectivity for all four dengue serotypes, compound 3-110-22, failed to inhibit stem-peptide binding in the competition assay (Figure 4). A likely explanation is that modification of the parental scaffold to produce 3-110-22 gave a compound with high affinity for the β-OG pocket in the E dimer, but lower affinity for the pocket-open state of the E trimer. The region that surrounds the mouth of the pocket differs in the two conformations, because of the fold-back of domain III (Figure 9), and 3-110-22 has a bulky substituent group. It appears that compound 3-110-22 indeed binds tightly to the E dimer, because unlike a number of others, it blocks the dimer-to trimer-transition of sE in vitro (Figure S4).

There are less likely alternative explanations for the inhibitory action of the compounds we have studied. One postulates a binding site, other than the β-OG pocket, that is present on monomeric DI/DII, on dimeric sE and on trimeric sE and that overlaps the peptide site on the trimer; another is a multi-site and multi-step mechanism. There is no evident candidate site for the former mechanism. The latter requires correlated affinities and properties of multiple sites. We therefore suggest that our peptide-competition, high-throughput screen has identified a large set of molecules that bind the β-OG pocket and that we have devised a useful assay for pocket-binding inhibitors, potentially applicable to any flavivirus for which one can prepare a stable, trimeric sE.

## Materials and Methods

### Peptide synthesis

Stem peptide 419–447, with the DV2 NGC sequence and an RGKGR solubility tag appended at its C-terminus, was synthesized using standard Fmoc chemistry on an ABI 431 Peptide Synthesizers at the Tufts University Core Facility (Boston, MA), purified using reverse phase HPLC, and analyzed by mass spectrometry. Fluorescein-isothiocyanate (FITC) was conjugated to the N-terminus of the peptide through a β-alanine linker.

### Liposomes

Liposomes [made with 1-palmitoyl-2-oleoyl-sn-glycero-3-phosphocholine (POPC), 1-palmitoyl-2-oleoyl-sn-glycero-3-phosphoethanolamine (POPE) (Avanti Polar Lipids) and cholesterol (Sigma-Aldrich) in a 1∶1∶2 molar ratio in TAN buffer (20 mM triethanolamine, 100 mM NaCl, pH 8.0)] were prepared by freeze-thaw extrusion through a 0.2 µ filter as described previously [24].

### Preparation of sE trimers

The postfusion sE trimer was produced as described [24]. Purified sE (Hawai'i Biotech) was incubated at 37°C in the presence of liposomes of the composition described above and acidified with MES buffer. Liposomes were solubilized with n-octyl-β-D-glucoside (β-OG) and n-undecyl-maltopyranoside (UDM). The solution was applied to a monoS column (GE Healthcare); sE trimer was eluted with a 2 M NaCl step gradient and further purified by size-exclusion chromatography on Superdex 200 (GE Healthcare). Protein was dialyzed extensively using a 50-KDa molecular-weight cutoff membrane (Spectrapor).

### Synthesis of 1662G07 analogs

Synthetic analogs of 1662G07 were prepared as described in Text S1 [33].

### High-throughput screening

All screens were preformed at the NSRB at Harvard Medical School. Binding experiments were carried out in Corning, low-volume 384 well microplates and analyzed in a *PerkinElmer EnVisions* instrument (excitation wavelength, 485 nm; emission wavelength, 535 nm). sE trimer was added to each well at 0.150 µM in 30 µL of TAN buffer. 0.1 µL of compound was transferred to each well and incubated at room temperature for 1 hr before the addition of DV2^419–447^ at a final concentration of 20 nM. After a 3-hour incubation, plates were read and fluorescence polarization measurements recorded. The original hit described in this paper came from the Maybridge 5 screening library at the NSRB at Harvard Medical School.

### Trypsin sensitivity (content-mixing) assay

This assay was essentially as described previously [24], [34]. Liposomes were made with added trypsin at 10 mg/ml. Unencapsulated trypsin was removed by passage of the suspension through a Superdex 200 gel filtration column (GE Healthcare). Small molecules and peptide prepared as DMSO stocks were diluted into 50 µL of TAN buffer in the presence of purified virions. Reactions were incubated at 37°C for 15 mins, before the addition of trypsin-loaded liposomes, acidified with MES pretitrated to reach a final pH of 5.5, and incubated at 37°C for 15 mins. Reactions were neutralized to pH 8.0 with 1 M TEA. Trypsin digestion proceeded for 1 hr at 37°C. Aliquots of the reaction were resuspended in SDS-loading buffer with 2 mM PMSF, incubated for 20 mins at 100°C, and analyzed by SDS-PAGE followed by immunoblotting with an anti-dengue core and anti-E antibody.

### Surface plasmon resonance

Experiments were performed in duplicate on a Biacore 3000 instrument. DI/DII protein was immobilized to a CM5 biosensor chip per manufacturer's instructions. All experiments were carried out at 25 C in HBS-EP buffer (10 mM HEPES, 150 mM NaCl 3 mM EDTA and 0.005% (vol/vol) P20 surfactant). Sensorgrams were obtained by passing over small molecules diluted in HBS-EP buffer at specified concentrations at a flow rate of 50 µL/min with a 2 minute association phase and 10 minute dissociation phase. The sensor surface was not regenerated between experiments. Identical injections over blank lanes without protein were used and subtracted from the data to account for background and nonspecific interactions with the biosensor chip.

### Cells and viruses

C6/36 cells were maintained in L-15 medium supplemented with 10% fetal bovine serum penicillin and streptomycin (Invitrogen). For viral plaque assays, BHK-21 cells were seeded (5×10^4^cells/well) in 24-well, treated tissue-culture plates in α-MEM supplemented pen/strep antibiotics, and 5% Fetal Bovine Serum (FBS). Cells were plated <12 hrs before use and stored at 37°C with 5% CO_2_.

Dengue virus serotype 2 New Guinea Clone (NGC) was adsorbed to confluent layers of C6/36 cells for 1 hr at 25°C with rocking every 15 mins. L-15 medium (Mediatech) was added, and cells were incubated at 25°C until syncytium formation was observed. The supernatant was clarified by centrifugation at 1600 RPM at 4°C and stored at −80°C.

### Plaque assay

BHK-21 cells were seeded as described above. Aliquots from infections were diluted in 10 fold dilutions in Earle's balanced salt solution (EBSS), and 100 µl of each dilution were added to cells. Plates were incubated for 1 hr at 37°C and rocked every 15 mins. Unadsorbed virus was removed by washing with 1 ml PBS, after which 1 ml of α-MEM supplemented with 2% carboxymethylcellulose (CMC), pen/strep antibiotics, HEPES and 2% FBS, was added to each well and incubated at 37°C for 4 days. The CMC overlay was aspirated, and cells were washed 2× with 1 mL PBS and stained with crystal violet.

### Plaque reduction inhibition assays

Virus supernatant was diluted in EBSS to a stock concentration that would allow for infection at MOI of 1, based on 50,000 seeded cells. Small molecules (or carrier) were added to the inoculum as indicated for each experiment. Cells were infected for 1 hr at 37°C with gentle rocking every 15 mins. Virus (or virus∶small-molecule mixtures) were washed from cells with 1 mL of PBS and overlay medium (α-MEM supplemented with HEPES, pen/strep antibiotics and 2% FBS) added. Plates were incubated at 37°C for 24 hrs. Aliquots of the supernatant were withdrawn and stored at −80°C.

### Cytotoxicity assay

BHK-21 cells were seeded at a density of 15,000 cells in a 96 well format. Compounds or vehicle were serially diluted in EBSS and 100 µl were transferred to each well. Plates were incubated at 37°C for 1 hr, media was aspirated and cells were washed 2× with 200 µl of PBS. 200 µl of α-MEM supplemented with pen/strep antibiotics, and 2% FBS was added and incubated for 24 hrs at 37°C. 20 uL of alamarBlue (Invitrogen) was added directly to each well and incubate for 2 hrs and read for absorbance at 570 nm.

## Supporting Information

Figure S1 Cross-inhibition of dengue serotypes by 1662G07 analogs. (A–D) Inhibition of DV1–4 serotypes by analogs.
(DOC)

Figure S2 Lack of inhibitory activity of 1662G07 analogs against Kunjin virus infection.
(DOC)

Figure S3 Production and characterization of DI/DII.
(DOC)

Figure S4 Inhibition of sE trimer formation and cofloatation with liposomes.
(DOC)

Figure S5 Sequence alignment of residues in the β-OG pocket.
(DOC)

Figure S6 *In silico* docking of small molecules.
(DOC)

Figure S7 The 1662G07 analogs do not inhibit vesicular stomatitis virus (VSV) infection.
(DOC)

Figure S8 Direct plaque assay of selected compounds from the 3–110 series.
(DOC)

Figure S9 WNV DI/DII does not reverse small-molecule inhibition of DV2.
(DOC)

Table S1 Broad structure activity relationship of 1662G07.
(DOC)

Table S2 Additional compounds from the 3–149 series.
(DOC)

Table S3 Additional compounds from the 3–110 series.
(DOC)

Text S1 Supplementary methods detailing the synthesis and characterization of 1662G07 analogs.
(DOC)
