# Correction: Small-Molecule Inhibitors of Dengue-Virus Entry

**DOI:** 10.1371/journal.ppat.1007553

**Published:** 2019-01-31

**Authors:** Aaron G. Schmidt, Kyungae Lee, Priscilla L. Yang, Stephen C. Harrison

The structure of compound 3-110-22 depicted in Figs 4–8, and the Supporting Files S2, S8 and S9 Figs is incorrect. A double-bond on the five-membered ring is missing. Please see the corrected Figs 4–8, and the Supporting Files S2, S8 and S9 Figs here.

**Fig 4:**
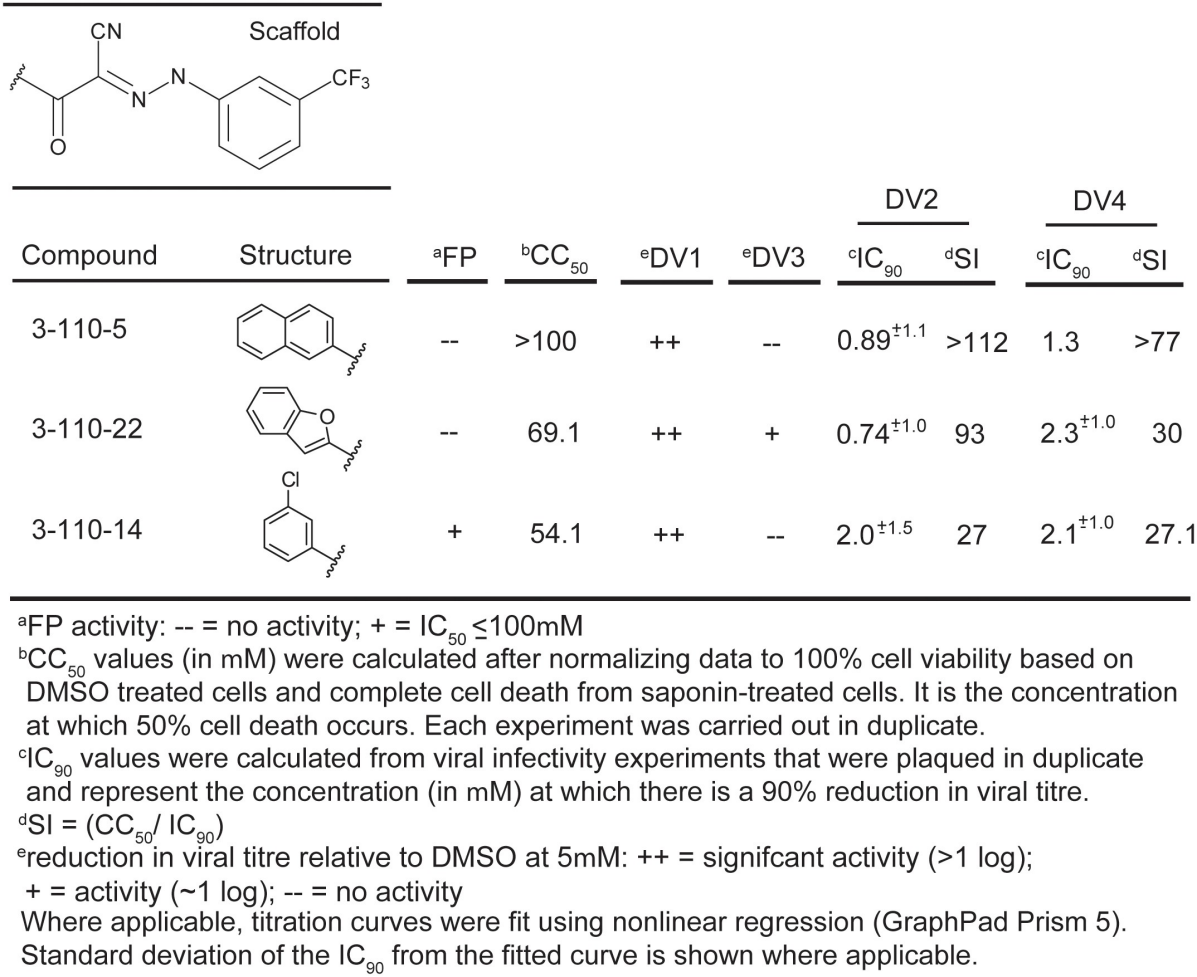
Biochemical, cytotoxicity and antiviral summary of selected compounds from the 3–110 series.

**Fig 5:**
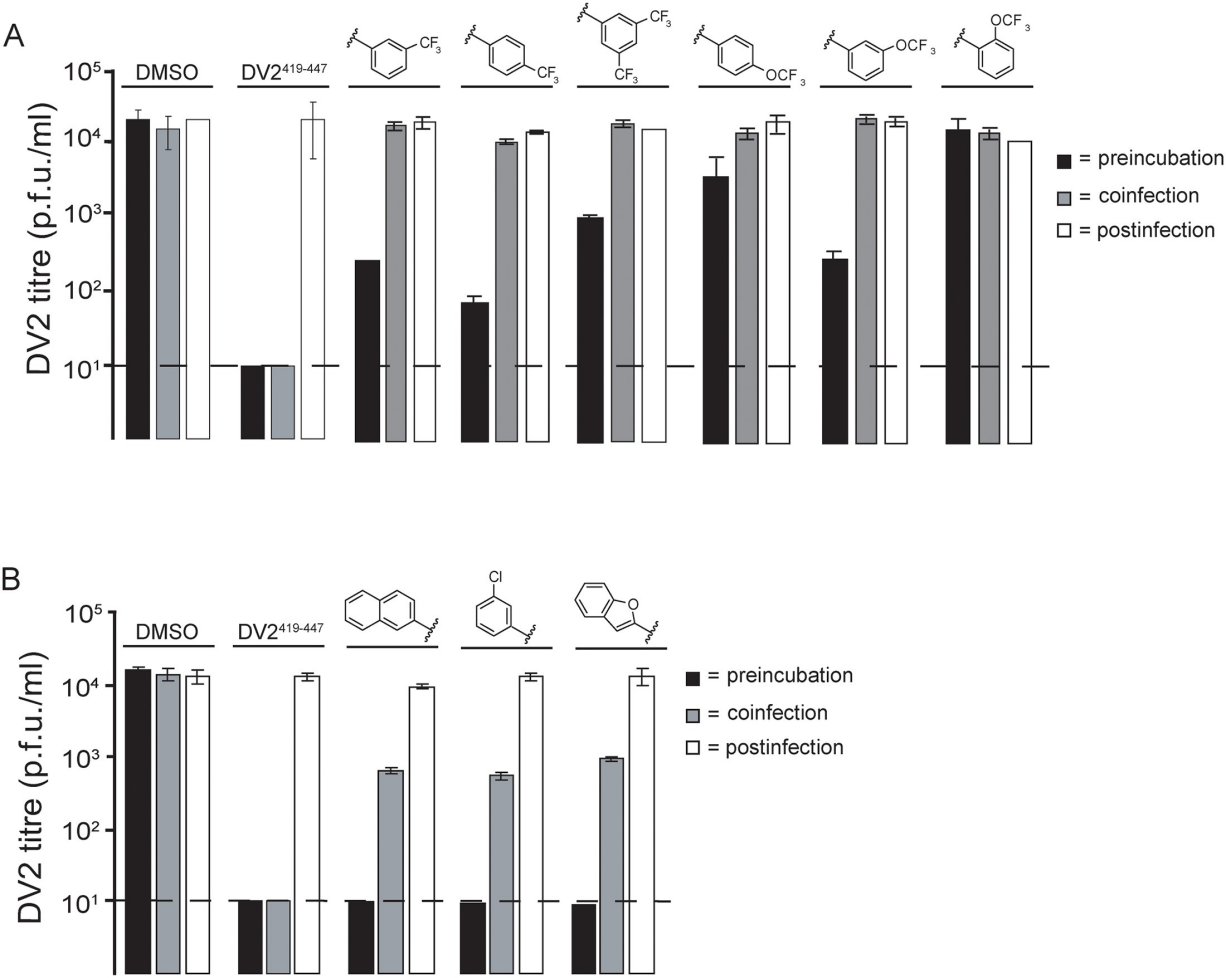
Effect of order-of-addition on small-molecule inhibition. (A) Comparison of *o*-, *m*-, and *p*-OCF_3_ and *m*-, di-*m*- and *p*-CF_3_ substitution from the 3–148 and 3–149 series (B) Comparison of compounds from the 3–110 series. Preincubation: addition of 1662G07 analogs to inoculum 15′ before adsorption to cells. Coinfection: addition of analogs at the time of adsorption. Postinfection: addition of analogs one hour after adsorption of virus. In all cases, cells were washed with PBS before adding compounds. Supernatants were harvested after 24 hours and viral titres determined by standard plaque forming assay (done in duplicate). Compounds from (A) and (B) were used at 15 and 5 μM, respectively. DV2^419–447^ stem peptide at 1 μM was used as a control.

**Fig 6:**
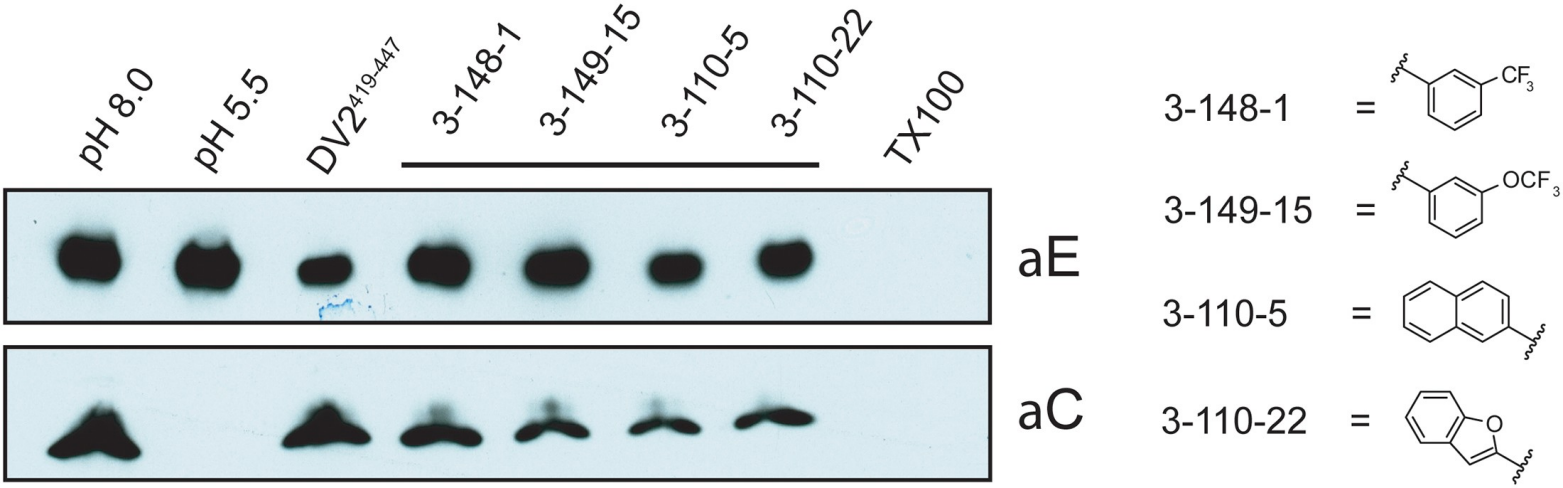
Inhibition of viral fusion with liposomes. Effect on content mixing of preincubating virus with 1662G07 analogs. Virus and analogs 3-148-1, 3-149-15, 3-110-5 and 3-110-22 (all at 50 μM) were incubated with liposomes encapsulating trypsin and acidified to pH = 5.5. Following back-neutralization and incubation for 1 hr at 37 C, samples were prepared for SDS-PAGE and immunoblotted with αC and αE antibody. Fusion leads to exposure of core protein to trypsin and loss of the corresponding band but retention of the envelope protein band. DV2^419–447^ stem peptide, at 1 μM, was used as a positive control.

**Fig 7:**
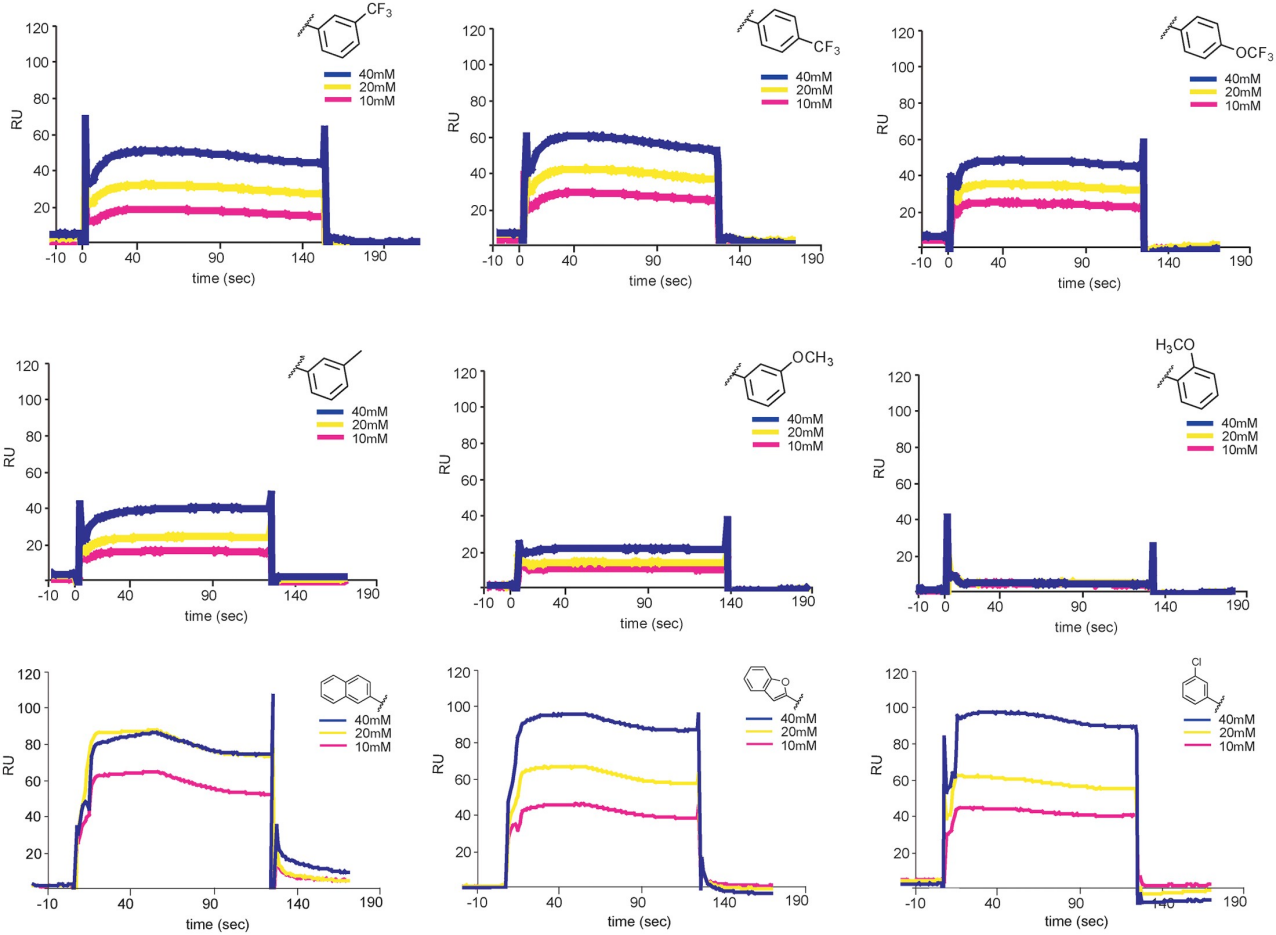
Interaction of 1662G07 analogs with DI/DII. DI/DII was immobilized on a CM5 sensorchip. Analogs 3-148-1, 3-149-3, 3-149-14, 3-151-2, 3-151-2, 3-151-5, 3-151-4, 3-110-5, 3-110-14 and 3-110-22 were passed over the DI/DII surface at 10, 20 and 40 μM. Background for nonspecific binding to the chip surface was corrected for by passing the analogs over a protein-free channel. All measurements carried out in duplicate.

**Fig 8:**
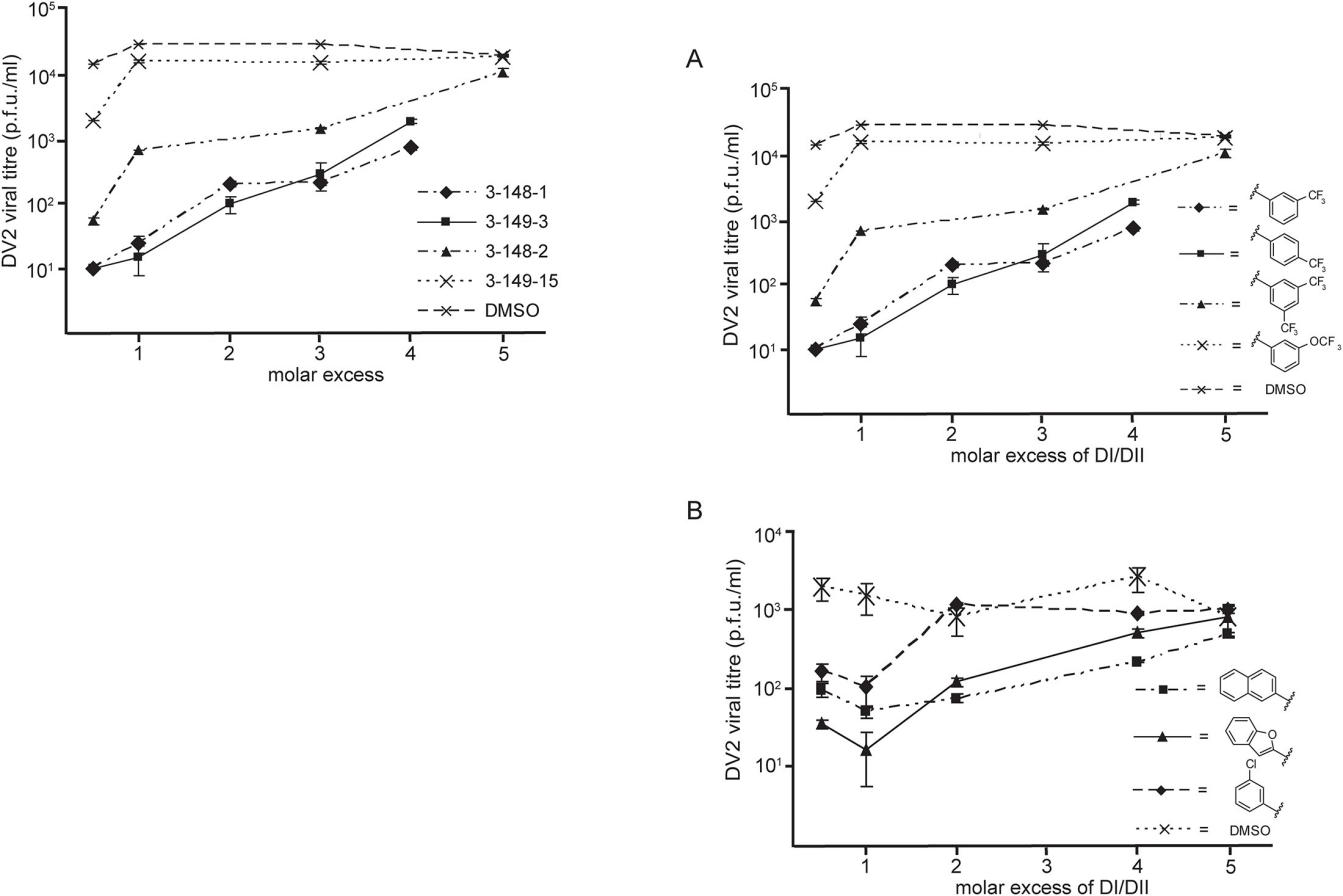
Reversibility of antiviral effect. Viral inocula were preincubated with 1662G07 analogs from the (A) 3–148 and 3–149 and (B) 3–110 series for 10′ at 37°C. DI/DII was then added in molar excess and the incubation continued for an additional 15′. Each inoculum was added to cells, and supernatants were harvested 24 hrs later. An inoculum preincubated with DI/DII alone at the same molar excess showed no loss in viral titre.

## Supporting information

S2 Fig Lack of inhibitory activity of 1662G07 analogs against Kunjin virus infection. (TIF)

S8 Fig Direct plaque assay of selected compounds from the 3–110 series. (TIF)

S9 Fig WNV DI/DII does not reverse small-molecule inhibition of DV2. (TIF)
