# Supplementary material for: Small-Molecule Inhibitors of Dengue-Virus Entry
Source: PLoS Pathog. 2012 Apr 5;8(4):e1002627. doi: 10.1371/journal.ppat.1002627 (PMC3320583; doi:10.1371/journal.ppat.1002627)
Supplement: Figure S1 — Cross-inhibition of dengue serotypes by 1662G07 analogs. (A–D) Inhibition of DV1–4 serotypes by analogs. (DOC) [file ppat.1002627.s001.doc]

**
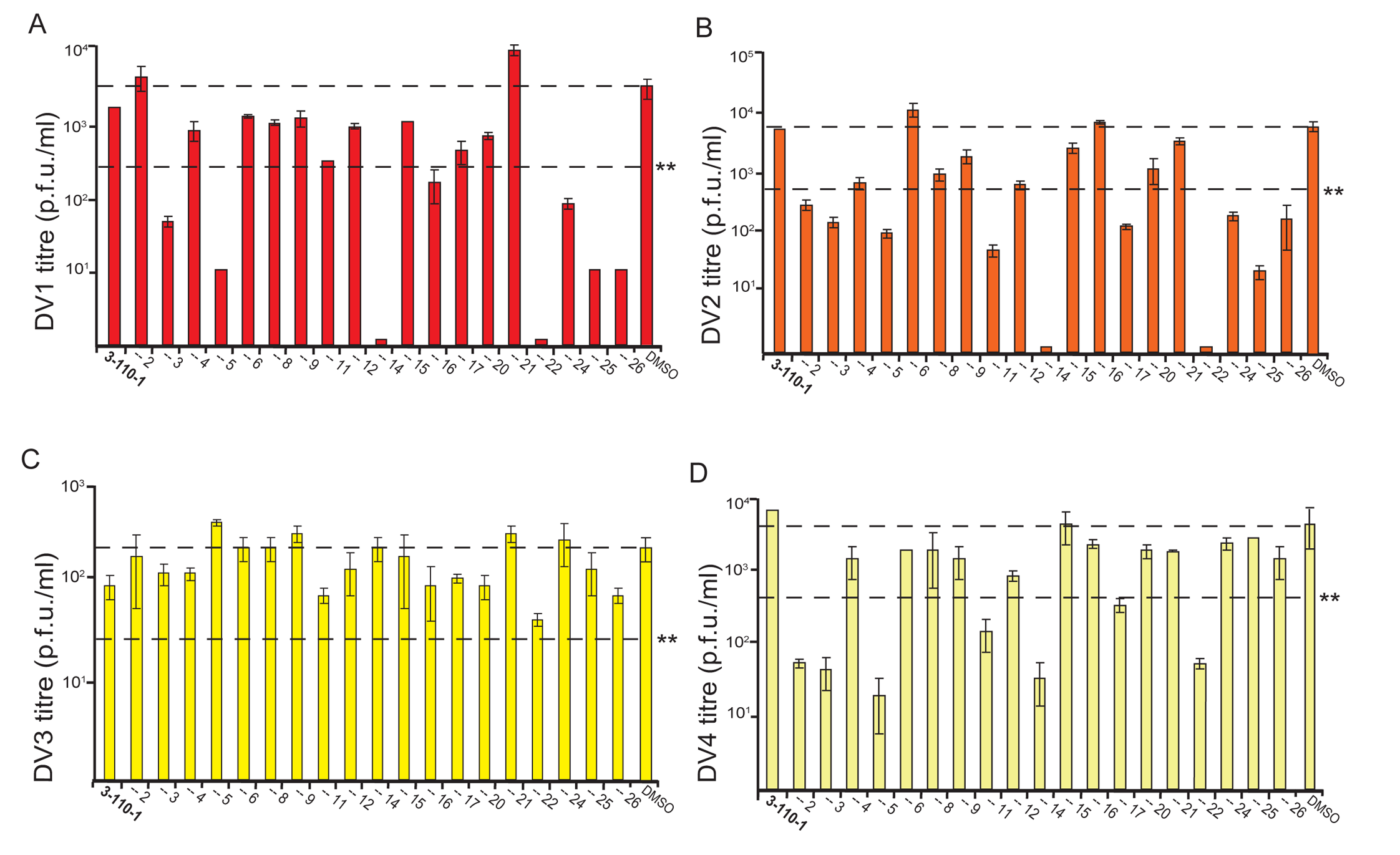
**

**Figure S1:** Cross-inhibition of dengue serotypes by 1662G07 analogs. (A-D) Inhibition of DV1-4 serotypes by analogs. Compounds were preincubated with virus inoculum and assayed by PFA or FFA as described in the text. Top dashed-line is the titre of the vehicle-treated control. Based on this, a second dashed-line denoted by **, represents the titre at which 90% reduction occurs. Viral infectivity for each concentration point was determined in duplicate. The numbering follows 3-110-xx, from Figure 4 and Table S3. Compounds were screened at 5μM and preincubated with the viral inoculum for 15 minutes before adsorption to cells.
