# Supplementary material for: Small-Molecule Inhibitors of Dengue-Virus Entry
Source: PLoS Pathog. 2012 Apr 5;8(4):e1002627. doi: 10.1371/journal.ppat.1002627 (PMC3320583; doi:10.1371/journal.ppat.1002627)
Supplement: Figure S2 — Lack of inhibitory activity of 1662G07 analogs against Kunjin virus infection. (DOC) [file ppat.1002627.s002.doc]

**
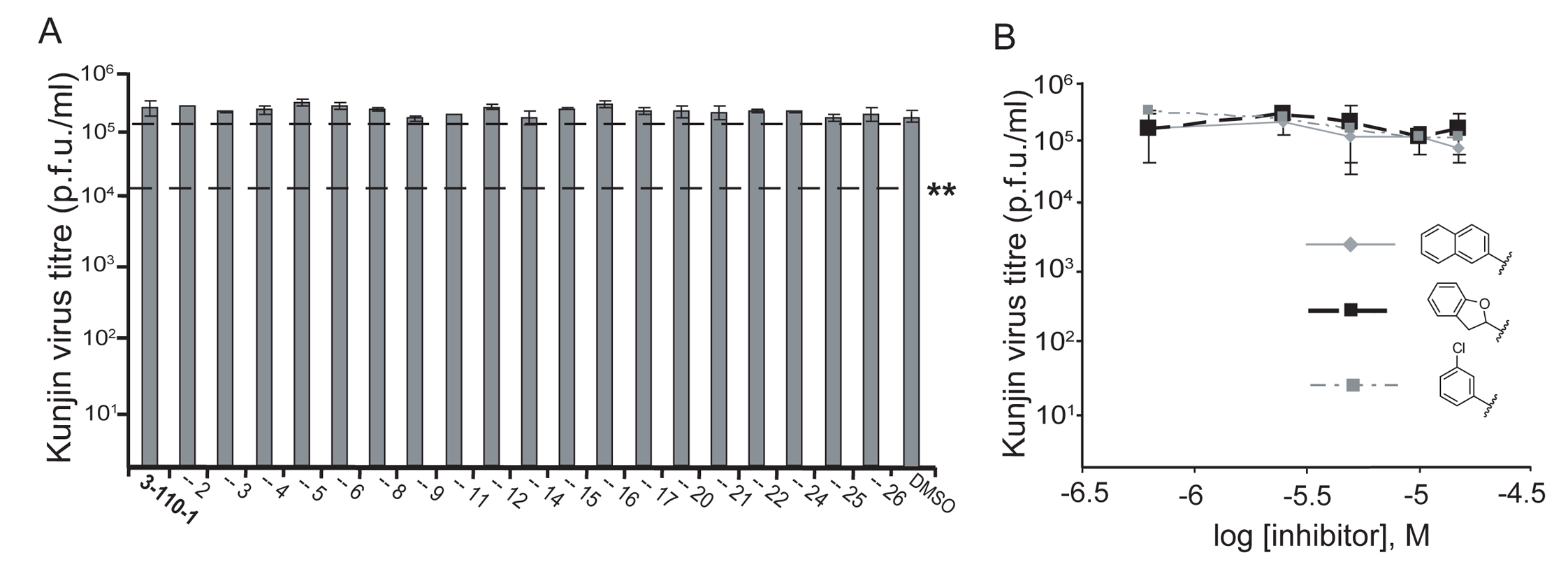
**

**Figure S2:** Lack of inhibitory activity of 1662G07 analogs against Kunjin virus infection. (A) 1662G07 analogs from Table S3 were tested at 5µM against Kunjin virus. Upper dashed-line is the titre of the vehicle-treated control. Lower dashed-line (labeled **) tire for 90% reduction occurs. (B) Effects of analogs from Figure 4 on Kunjin virus infectivity determined in duplicate for each concentration point.
