# Supplementary material for: Small-Molecule Inhibitors of Dengue-Virus Entry
Source: PLoS Pathog. 2012 Apr 5;8(4):e1002627. doi: 10.1371/journal.ppat.1002627 (PMC3320583; doi:10.1371/journal.ppat.1002627)
Supplement: Figure S3 — Production and characterization of DI/DII. (DOC) [file ppat.1002627.s003.doc]

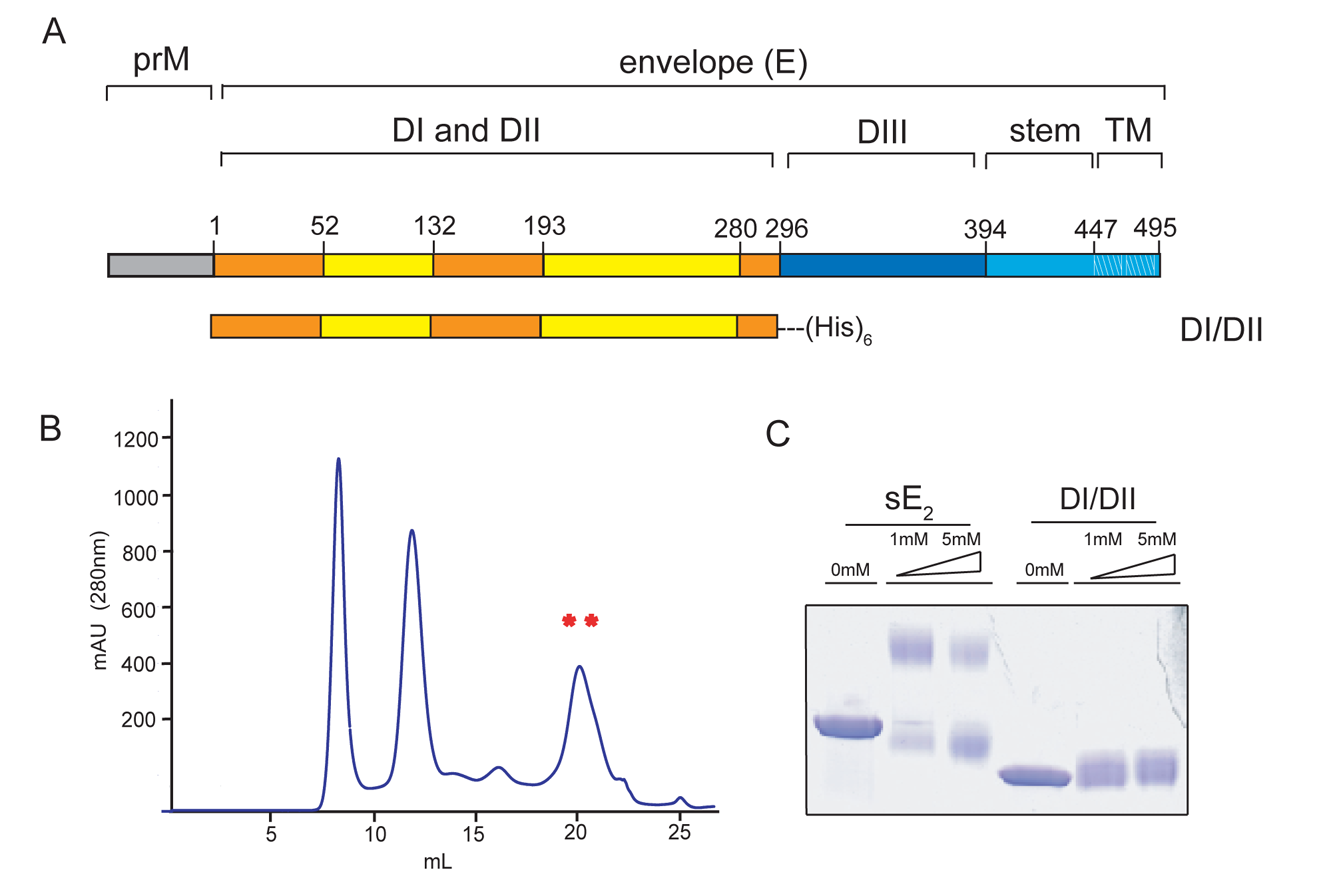


**Figure S3:** Production and characterization of DI/DII. (A) Linear schematic of full-length E. Domain I (orange), Domain II (yellow) and Domain III (blue) with the stem region (light blue) followed by the double-spanning transmembrane segment (light blue with white lines). The DI/DII construct was cloned from DV2 NGC cDNA (residues 1-295) with a C-terminal hexa-histidine tag using ligation independent cloning into the pFastBac vector for use in the Bac-to-Bac system (Invitrogen). The insert, which followed a signal sequence, was transformed into DH10Bac cells creating the recombinant bacmid. The bacmid was transfected into Sf21 cells. The viral stock was amplified and the resultant P3 was used to infect Hi-5 cells. DI/DII was harvested from the media by metal affinity chromatography using Ni-NTA resin (Qiagen) and further purified over a Superdex 200 column (GE-Healthcare). DI/DII was confirmed using SDS-PAGE and immunoblotting with conformation-specific monoclonal antibody 4G2 . This is construct is shown on a separate line. (B) Size-exclusion chromatography of DI/DII construct. Interaction of purified DI/DII construct (red asterisks) with the chromatography resin leads to a delayed elution volume. (C) Chemical crosslinking of DI/DII construct. 10µg of DI/DII was incubated with EGS for 30` at room temperature. Reactions were quenched by addition of 100mM TRIS and samples were analyzed by SDS-PAGE and stained with Coomassie Brilliant Blue; recombinant sE dimer was used control.
