# Supplementary material for: Small-Molecule Inhibitors of Dengue-Virus Entry
Source: PLoS Pathog. 2012 Apr 5;8(4):e1002627. doi: 10.1371/journal.ppat.1002627 (PMC3320583; doi:10.1371/journal.ppat.1002627)
Supplement: Figure S4 — Inhibition of sE trimer formation and cofloatation with liposomes. (DOC) [file ppat.1002627.s004.doc]

**
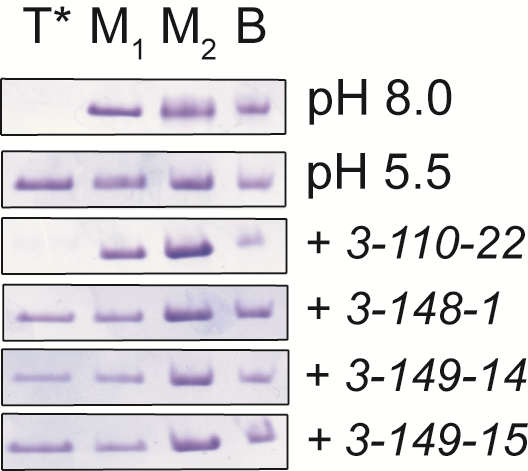
**

**Figure S4:** Inhibition of sE trimer formation and cofloatation with liposomes. Purified recombinant E protein (20µg) was preincubated with small molecule inhibitors at 10-fold molar excess over the protein (20µM final concentration) for 15 minutes at 37ºC. Liposomes were added (see Materials and Methods) and the reaction acidified to pH 5.5 or maintained at pH 8.0 for 10 minutes. The reactions were back-neutralized and adjusted to 25% sucrose (w/v). This protein:liposome solution was layered over a 40% sucrose solution, and a final 5% sucrose solution was added above it. The gradients were centrifuged in a Beckman SW60ti rotor at 215,000 X g at 4ºC for 3hrs. The top (T*), liposome-containing fraction was removed and the remaining gradient separated into three additional fractions (M_1_, M_2_ and B). sE associates stably with liposomes only under acidic conditions as a result of conversion of sE dimers to sE trimers. Compound 3-110-22 blocked trimer formation; compound 3-149-14 had a small inhibitory effect.
