# Supplementary material for: Small-Molecule Inhibitors of Dengue-Virus Entry
Source: PLoS Pathog. 2012 Apr 5;8(4):e1002627. doi: 10.1371/journal.ppat.1002627 (PMC3320583; doi:10.1371/journal.ppat.1002627)
Supplement: Figure S5 — Sequence alignment of residues in the β-OG pocket. (DOC) [file ppat.1002627.s005.doc]

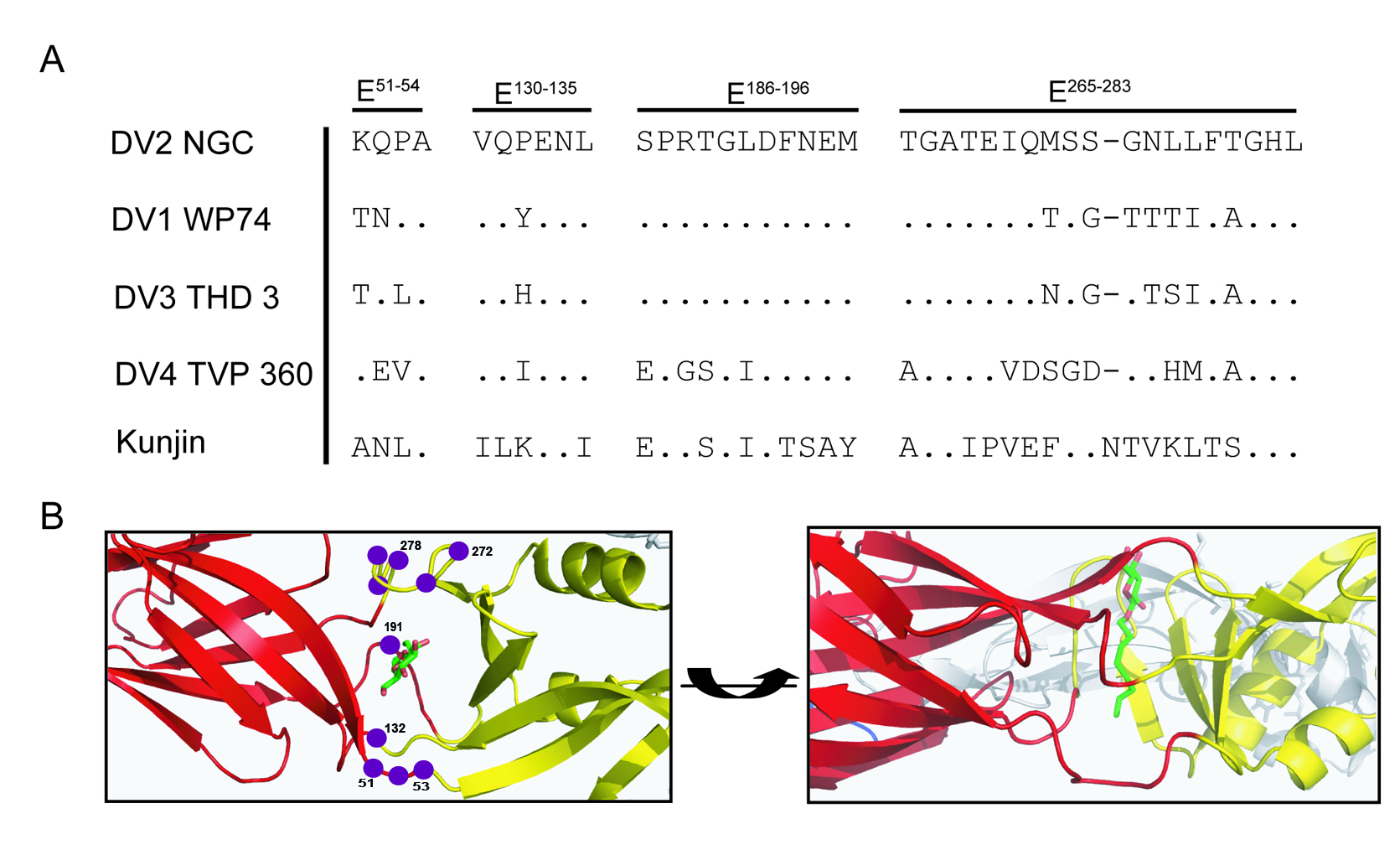


**Figure S5:** Sequence alignment of residues in the β-OG pocket. (A) Envelope residues that line the β-OG pocket are numbered based on the DV2 NGC sequence. Conservations among the dengue serotypes, Kunjin and WNV (NY99) are represented by “.” (B) Mapping of the least-conserved residues (purple dots) on the structure of the prefusion form of the dengue envelope protein with bound β-OG (PDB: 1OK8). Kunjin and WNV (NY99) deviate much more from the dengue consensus in this region than the dengue serotypes deviate from one another.
