# Supplementary material for: Small-Molecule Inhibitors of Dengue-Virus Entry
Source: PLoS Pathog. 2012 Apr 5;8(4):e1002627. doi: 10.1371/journal.ppat.1002627 (PMC3320583; doi:10.1371/journal.ppat.1002627)
Supplement: Figure S6 — In silico docking of small molecules. (DOC) [file ppat.1002627.s006.doc]

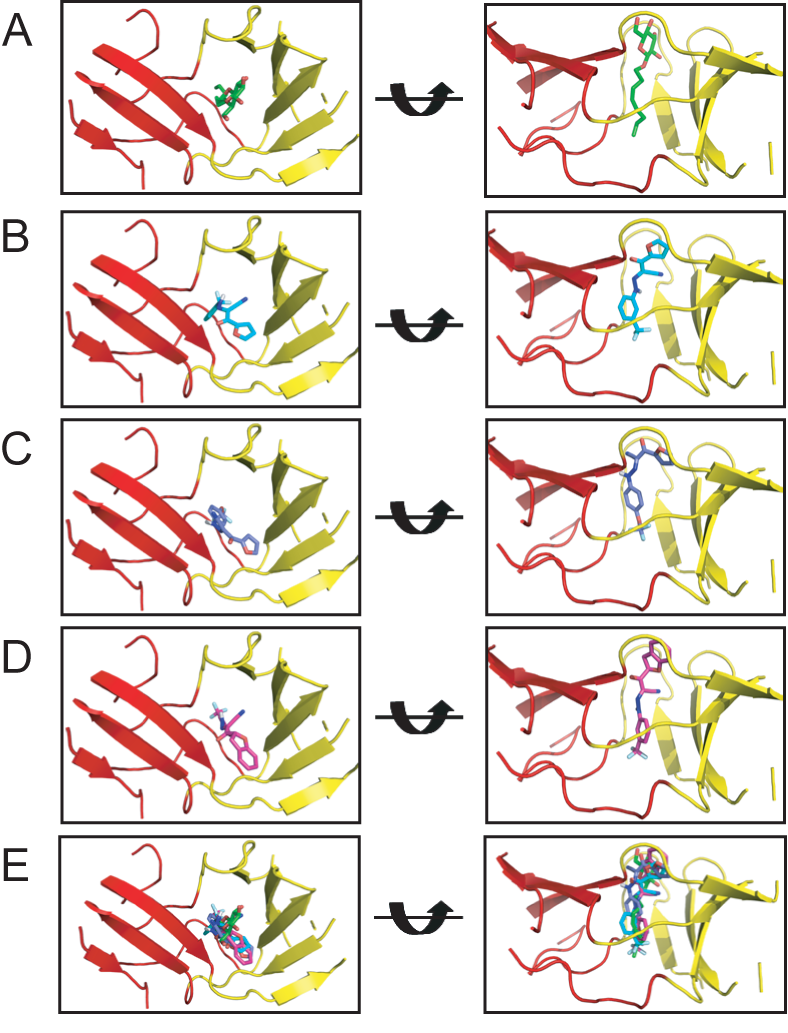


**Figure S6:** *In silico* docking of small molecules. Coordinates for selected compounds were prepared using LigPrep (Schrödinger) and docked into the prefusion form of the dengue envelope protein (PDB: 1OKE) using GLIDE [1, 2]. Panels show “top” and “side” views of (A) β-OG from the crystal structure, (B) parental compound 1662G07, (C) compound 3-149-14, (D) compound 3-110-22, each docked into the β -OG pocket depicted in Fig S5. (E) Superposition of all three compounds with β -OG.

**REFERENCES**

1. Friesner RA, Banks JL, Murphy RB, Halgren TA, Klicic JJ, et al (2004) Glide: A New Approach for Rapid, Accurate Docking and Scoring. 1. Method and Assessment of Docking Accuracy. J Med Chem 47: 1739-1749.
2. Friesner RA, Murphy RB, Repasky MP, Frye LL, Greenwood JR, et al (2006) Extra Precision Glide: Docking and Scoring Incorporating a Model of Hydrophobic Enclosure for Protein-Ligand Complexes. J Med Chem 49: 6177-6196.
