# Supplementary material for: Small-Molecule Inhibitors of Dengue-Virus Entry
Source: PLoS Pathog. 2012 Apr 5;8(4):e1002627. doi: 10.1371/journal.ppat.1002627 (PMC3320583; doi:10.1371/journal.ppat.1002627)
Supplement: Figure S7 — The 1662G07 analogs do not inhibit vesicular stomatitis virus (VSV) infection. (DOC) [file ppat.1002627.s007.doc]

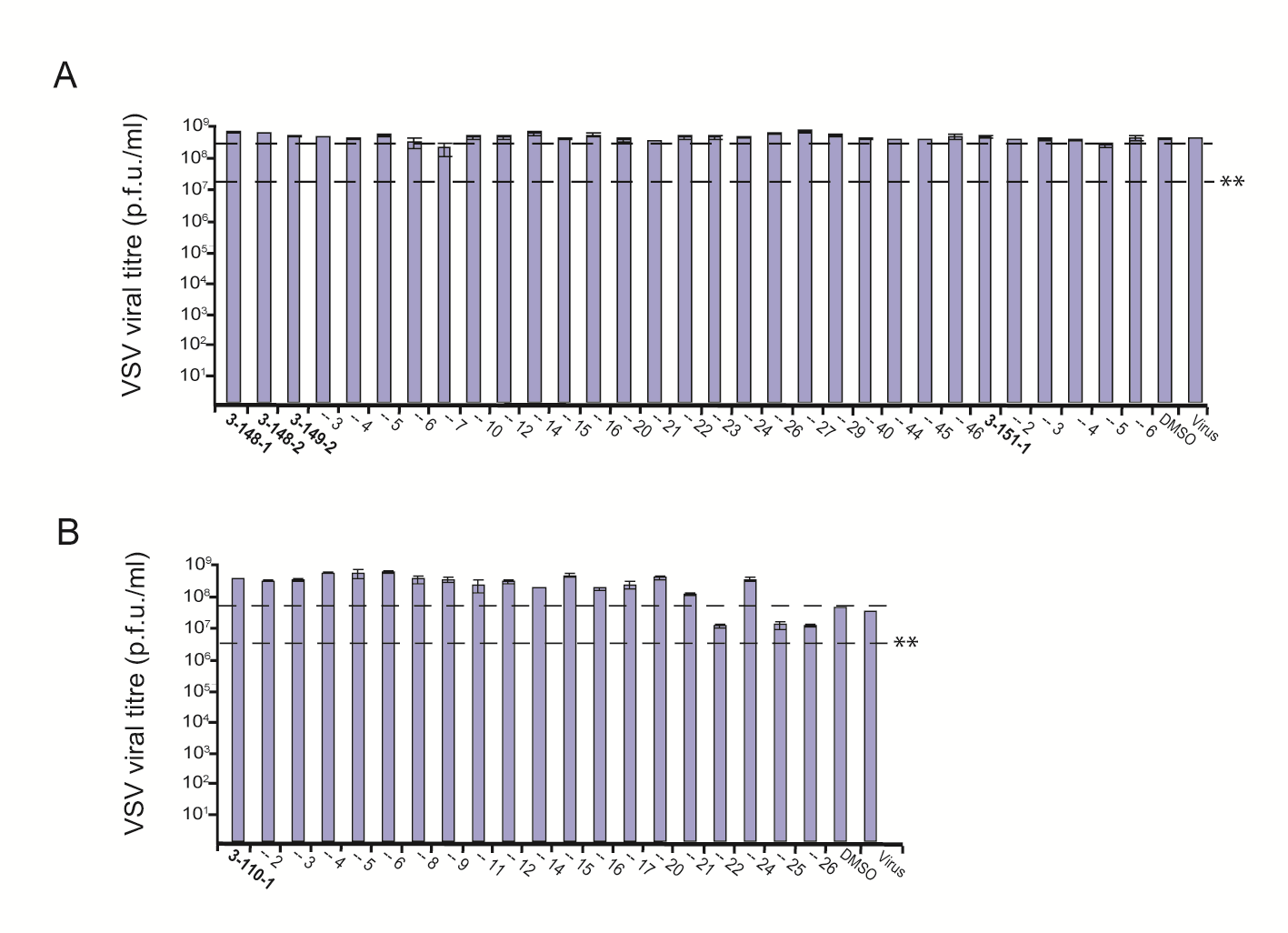


**Figure S7:** The 1662G07 analogs do not inhibit vesicular stomatitis virus (VSV) infection. (A) 1662G07 analogs from the 3-148, 3-149 and 3-151 series tested at 20μM. Upper dashed line, titre of the vehicle-treated control; lower dashed line (labeled **), titre for 90% reduction. (B) Effects of analogs from Figure 4 and Table S3 on VSV infectivity determined in duplicate for each concentration point.
