# Supplementary material for: Small-Molecule Inhibitors of Dengue-Virus Entry
Source: PLoS Pathog. 2012 Apr 5;8(4):e1002627. doi: 10.1371/journal.ppat.1002627 (PMC3320583; doi:10.1371/journal.ppat.1002627)
Supplement: Figure S8 — Direct plaque assay of selected compounds from the 3–110 series. (DOC) [file ppat.1002627.s008.doc]

**
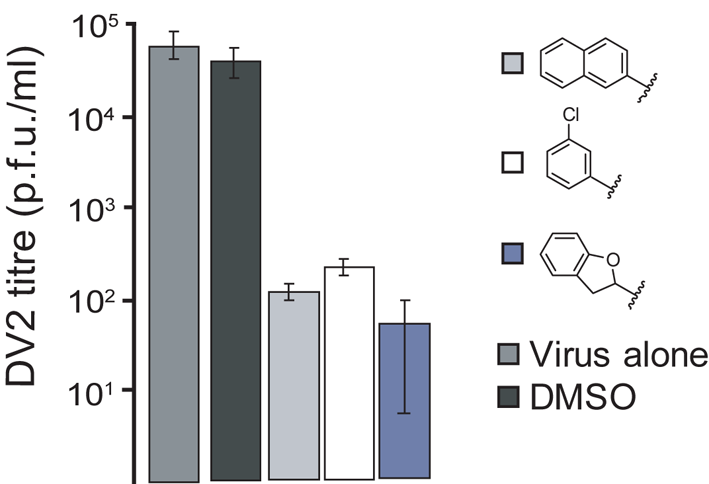
**

**Figure S8:** Direct plaque assay of selected compounds from the 3-110 series. Briefly, ~50,000 pfu of DV2 were preincubated with compounds (5µM) for 10` at 37ºC and adsorbed to BHK cells for 1hr at 37ºC. Monolayers were washed twice with PBS, overlayed with CMC and incubated at 37ºC for 4 days. Plates were developed as described in Methods. Each experiment was preformed in duplicate.
