# Supplementary material for: Small-Molecule Inhibitors of Dengue-Virus Entry
Source: PLoS Pathog. 2012 Apr 5;8(4):e1002627. doi: 10.1371/journal.ppat.1002627 (PMC3320583; doi:10.1371/journal.ppat.1002627)
Supplement: Figure S9 — WNV DI/DII does not reverse small-molecule inhibition of DV2. (DOC) [file ppat.1002627.s009.doc]

**
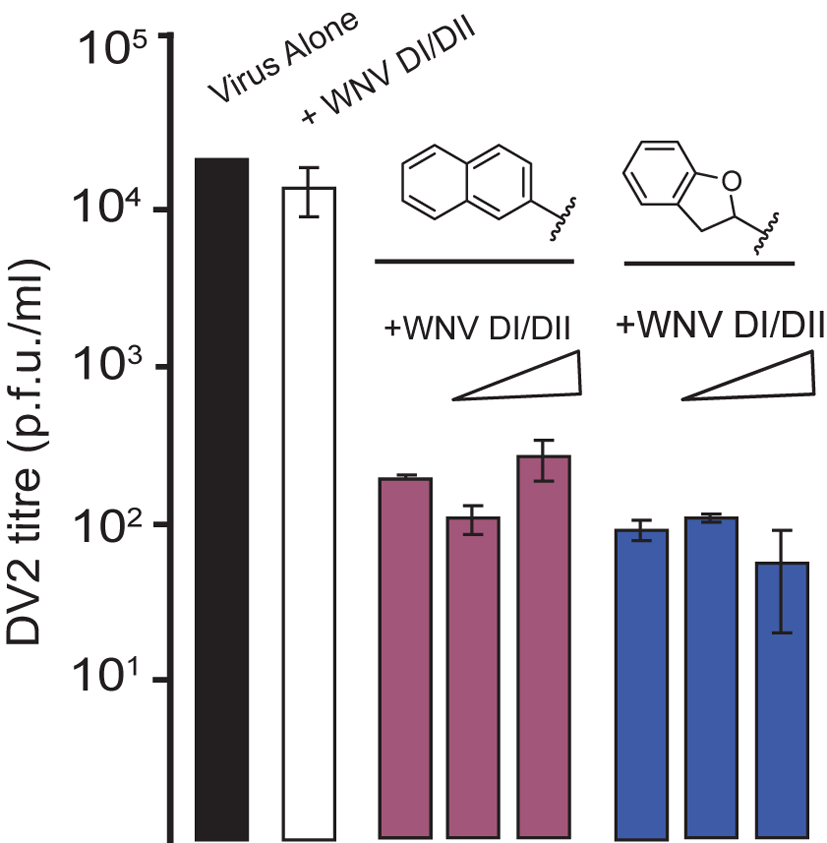
**

**Figure S9:** WNV DI/DII does not reverse small-molecule inhibition of DV2. WNV DI/DII (residues 1-295, NY99 strain) was cloned into a mammalian cell expression plasmid with a C-terminal hexa-histidine tag and a TPA signal sequence (See Supplementary Fig. 5 for sequence comparison of Kunjin and WNV (NY99) surrounding the β-OG pocket. 293T cells were transfected with WNV DI/DII DNA using Lipofectamine 2000 (Invitrogen) following manufacturer’s protocol. Cell supernatant was harvested 48hrs later and purified as described above for DV2 DI/DII. Reactivity with conformational specific antibody 4G2 was used to monitor expression and purification. Reversibility experiments were performed as described in Figure 8. Briefly, select compounds from the 3-110 series were preincubated with DV2 inoculum for 10’ at 37ºC. WNV DI/DII was then added at 1 and 5 molar excess of small molecule and incubated for an additional 10’ at 37ºC. Viral titres were determined by standard PFA as described in Methods.
