# Supplementary figures and images for: Small-Molecule Inhibitors of Dengue-Virus Entry
Source: PLoS Pathog. 2012 Apr 5;8(4):e1002627. doi: 10.1371/journal.ppat.1002627 (PMC3320583; doi:10.1371/journal.ppat.1002627)

**Table S1**

**
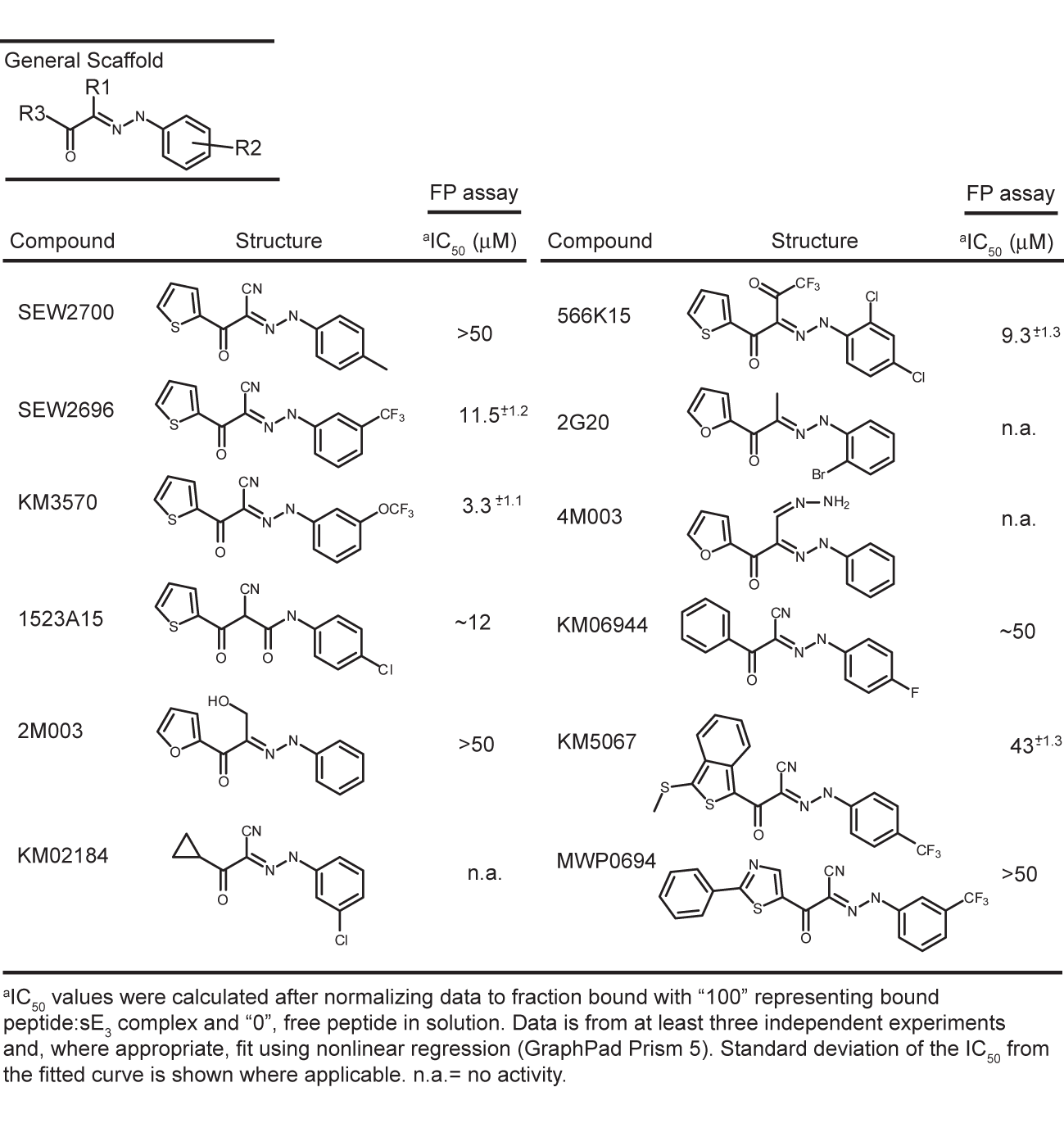
**

Supplement: Table S1 — Broad structure activity relationship of 1662G07. (DOC) [file ppat.1002627.s010.doc]

**Table S2**

**
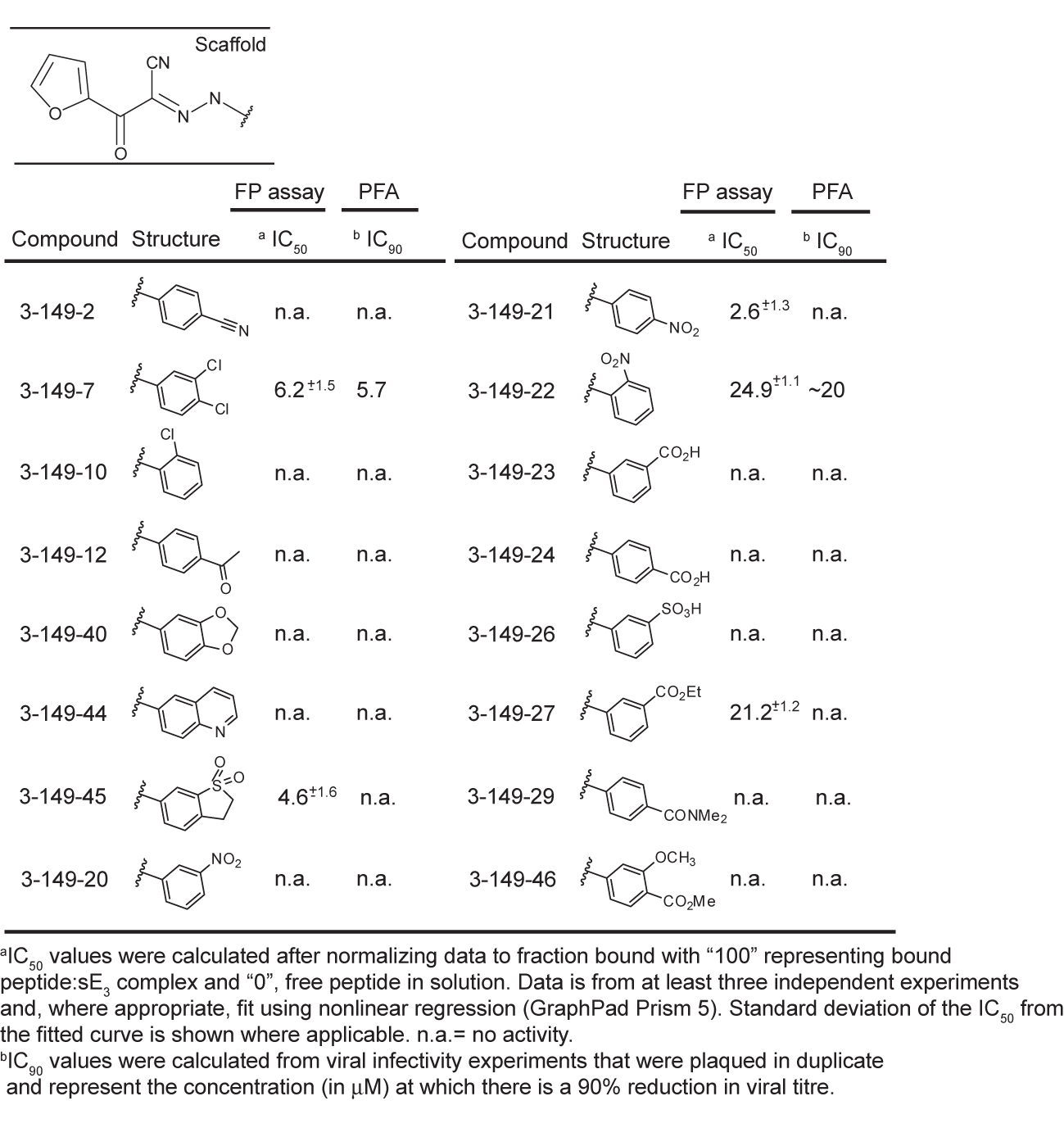
**

Supplement: Table S2 — Additional compounds from the 3–149 series. (DOC) [file ppat.1002627.s011.doc]

**Table S3**


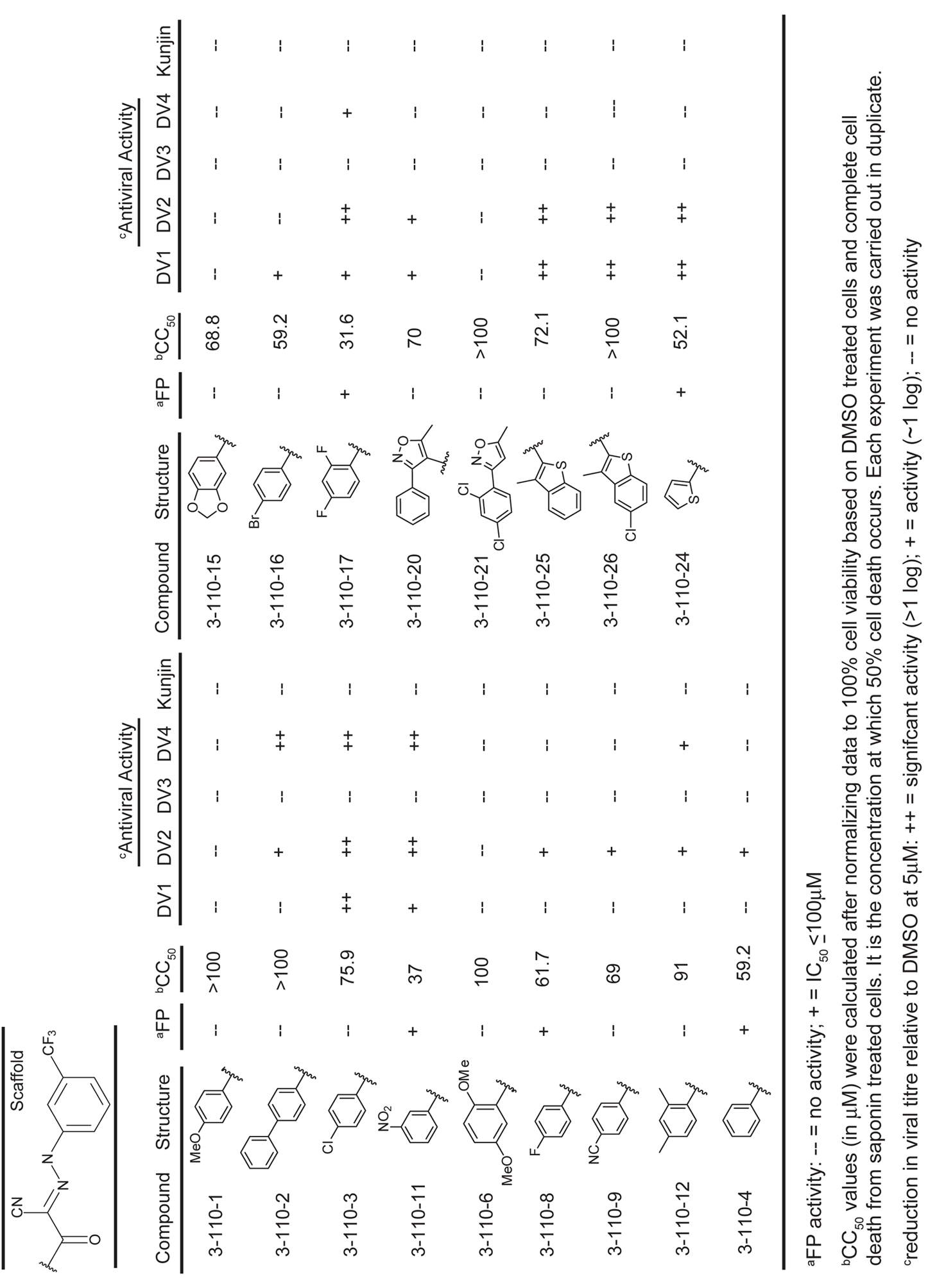

Supplement: Table S3 — Additional compounds from the 3–110 series. (DOC) [file ppat.1002627.s012.doc]
