# Supplementary material for: Small-Molecule Inhibitors of Dengue-Virus Entry
Source: PLoS Pathog. 2012 Apr 5;8(4):e1002627. doi: 10.1371/journal.ppat.1002627 (PMC3320583; doi:10.1371/journal.ppat.1002627)
Supplement: Text S1 — Supplementary methods detailing the synthesis and characterization of 1662G07 analogs. (DOC) [file ppat.1002627.s013.doc]

**Text S1**

**Supplementary Methods**

Small-molecule inhibitors of dengue-virus entry

Aaron G. Schmidt^1^, Kyungae Lee^2^, Priscilla L. Yang^3^, Stephen C. Harrison^1,4^

^1^. Jack and Eileen Connors Laboratory of Structural Biology, Department of Biological Chemistry and Molecular Pharmacology, Harvard Medical School, Boston, Massachusetts, United States of America, ^2^. New England Regional Center of Excellence in Biodefense and Emerging Infectious Diseases (NERCE/BEID), Harvard Medical School, Boston, Massachusetts, United States of America, and ^3^. Department of Microbiology and Molecular Genetics, Harvard Medical School, Boston, Massachusetts, United States of America, ^4.^Howard Hughes Medical Institute Harvard Medical School, Boston, Massachusetts, United States of America.

**Supplementary Methods**

1. General information
2. Representative synthesis of 3-148 series and characterization data
3. Parallel synthesis of 3-110 series and MS characterization data

**I. General information**

^1^H NMR spectra were recorded on a Varian Inova 600 MHz spectrometer with chemical shifts reported in parts per million (ppm) relative to an internal standard (trimethylsilane). Coupling constants (*J*) are reported in hertz (Hz). Standard resolution mass spectra were obtained on an Agilent 1200 Series HPLC (4.6 x 100 mm, 5 µm Phenomenex C18 reverse-phase column) and a 6130 Series mass spectrometer system; all mass spectra were obtained using electrospray ionization (EI) in positive ion mode. Standard reverse-phase HPLC conditions were as follows: mobile phase A = 0.1% formic acid in water; mobile phase B = 0.1% formic acid in acetonitrile. Solvents for synthesis were purchased as anhydrous grade and used without further purification. Reagents were purchased from commercial sources and used as received.

**II. Representative synthesis of 3-148 series** (**compound 3-148-1) and characterization data**

To a solution of 3-(trifluoromethyl)aniline (37 uL, 0.30 mmol) in 6-N hydrochloric acid (1mL) was added dropwise a solution of sodium nitrite (21 mg, 0.30 mmol) in water (1mL) at 5 ^o^ C. After stirring for 15 min at ambient temperature, a mixture of 2-furoylacetonitrile (34 mg, 0.25 mmol) and sodium acetate (123 mg, 1.5 mmol) in dioxane (2 mL) was slowly added and the resulting mixture was stirred for 4 hrs at ambient temperature, diluted with water (5 mL), and extracted with ethyl acetate. The combined extracts were washed with saturated brine, dried over magnesium sulfate, and concentrated in vacuo. The residue was purified by flash chromatography on SiO_2_ (47mg, 61 % yield). ESI-MS (m/z)= 308.0; ^1^H NMR (600 MHz, CDCl_3_) δ 7.91 (d, 1H, *J*=3.6), 7.80 (d, 1H, *J*=1.8), 7.71 (s, 1H), 7.62-7.55 (m, 2H), 7.50 (d, 1H, *J*=7.8), 6.68 (dd, 1H, *J*=3.6, 1.8).

3-148-2: ESI-MS (m/z)= 376.0; ^1^H NMR (600 MHz, CDCl_3_) δ 9.43 (br s, 1H), 7.95 (d, 1H, *J*=4.2), 7.86 (s, 1H), 7.84 (s, 1H), 7.79 (s, 1H), 7.73 (s,1H), 6.70 (dd, 1H, *J*=3.6, 1.8).

3-149-3: ESI-MS (m/z)= 308.0; ^1^H NMR (600 MHz, CDCl_3_) δ 9.36 (br s, 1H), 7.78 (s, 1H), 7.73 (d, 2H, *J=*8.4), 7.61 (d, 1H, *J=*3.6), 7.43 (d, 2H, *J=*8.4), 6.66 (dd, 1H, *J*=3.6, 1.8).

3-149-4: ESI-MS (m/z)= 319.1; ^1^H NMR (600 MHz, CDCl_3_) δ 7.91 (d, 1H, *J*=4.2), 7.84 (d, 1H, *J*=7.8), 7.79 (d, 1H, *J*=1.2), 7.60 (d, 1H, *J*=7.8), 7.42 (t, 1H, *J*=7.8), 7.13 (t, 1H, *J*=7.2), 6.67 (dd, 1H, *J*=4.2, 1.8).

3-149-5: ESI-MS (m/z)= 319.1; ^1^H NMR (600 MHz, CDCl_3_) δ 7.89 (d, 1H, *J*=3.6), 7.78 (d, 1H, *J*=1.2), 7.66 (s, 1H), 7.39-7.29 (m, 3H), 6.66 (dd, 1H, *J*=3.6, 1.2).

3-149-6: ESI-MS (m/z)= 319.1; ^1^H NMR (600 MHz, CDCl_3_) δ 7.88 (d, 1H, *J*=3.6), 7.78 (d, 1H, *J*=1.2), 7.56 (d, 2H, *J*=9), 7.33 (d, 2H, *J*=9), 6.67 (dd, 1H, *J*=3.6, 1.8).

3-149-14: ESI-MS (m/z)= 324.0; ^1^H NMR (600 MHz, CDCl_3_) δ 7.89 (d, 1H, *J*=3.6), 7.78 (d, 1H, *J*=1.2), 7.48 (d, 2H, *J*=9), 7.30 (d, 2H, *J*=9), 6.54 (dd, 1H, *J*=3.6, 1.8).

3-149-15: ESI-MS (m/z)= 324.0; ^1^H NMR (600 MHz, CDCl_3_) δ 9.42 (br s, 1H), 7.90 (d, 1H, *J*=4.2), 7.79 (s, 1H), 7.58 (d, 1H, *J*=3.6), 7.49-7.43 (m, 1H), 7.36-7.28 (m, 2H), 6.68 (dd, 1H, *J*=4.2, 1.8).

3-149-16: ESI-MS (m/z)= 324.0; ^1^H NMR (600 MHz, CDCl_3_) δ 7.91 (d, 1H, *J*=3.6), 7.90-7.89 (m, 1H), 7.79 (d, 1H, *J*=1.8), 7.40 (t, 1H, *J*=8.4), 7.34 (d, 1H, *J*=8.4), 7.28-7.25 (m, 1H), 6.67 (dd, 1H, *J*=3.6, 1.8).

3-151-1: ESI-MS (m/z)= 254.1; ^1^H NMR (600 MHz, CDCl_3_) δ 9.32 (br s, 1H), 7.76 (d, 1H, *J*=1.2), 7.64 (d, 1H, *J=*3), 7.53 (d, 1H, *J=*8.4), 7.34 (t, 1H, *J*=7.8), 7.18 (d, 1H, *J=*8.4), 7.16 (t, 1H, *J*=7.8), 6.64 (dd, 1H, *J*=3.6, 1.8), 2.42 (s, 3H).

3-151-2: ESI-MS (m/z) = 254.1; ^1^H NMR (600 MHz, CDCl_3_) δ 7.86 (d, 1H, *J*=3.6), 7.76 (d, 1H, *J=*1.2), 7.35-7.29 (m, 2H), 7.22 (d, 1H, *J*=7.8), 7.07 (d, 1H, *J=*7.2), 6.65 (dd, 1H, *J*=3.6, 1.8), 2.40 (s, 3H).

3-151-3: ESI-MS (m/z)= 254.1; ^1^H NMR (600 MHz, CDCl_3_) δ 9.33 (br s, 1H), 7.74 (d, 1H, *J*=1.8), 7.61 (d, 1H, *J=*3.6), 7.27-7.23 (m, 4H), 6.63 (dd, 1H, *J*=3.6, 1.8), 2.39 (s, 3H).

3-151-4: ESI-MS (m/z)= 270.1; ^1^H NMR (600 MHz, CDCl_3_) δ 9.89 (br s, 1H), 7.75 (d, 1H, *J*=1.8), 7.63 (d, 1H, *J=*3.6), 7.53 (d, 1H, *J=*7.8), 7.19 (t, 1H, *J=*7.2), 7.07 (t, 1H, *J=*7.2), 7.0 (d, 1H, *J=*8.4), 6.63 (dd, 1H, *J*=3.6, 1.8), 3.95 (s, 3H).

3-151-5: ESI-MS (m/z)= 270.1; ^1^H NMR (600 MHz, CDCl_3_) δ 9.29 (br s, 1H), 7.75 (d, 1H, *J*=1.8), 7.62 (d, 1H, *J=*3.6), 7.36 (t, 1H, *J=*8.4), 6.92-6.89 (m, 2H), 6.78 (dd, 1H, *J=*8.4, 2.4), 6.63 (dd, 1H, *J*=3.6, 1.8), 3.85 (s, 3H).

3-151-6: ESI-MS (m/z)= 270.1; ^1^H NMR (600 MHz, CDCl_3_) δ 9.32 (br s, 1H), 7.78 (d, 1H, *J*=1.8), 7.59 (d, 1H, *J*=3.6), 7.30 (d, 2H, *J*=8.4), 6.99 (d, 2H, *J*=8.4), 6.61 (dd, 1H, *J*=3.6, 1.8), 3.82 (s, 3H).

**III.** **Parallel synthesis of 3-110 series** **and MS characterization data**

To a solution of 3-(trifluoromethyl)aniline (0.45 mL, 3.6 mmol) in 6-N hydrochloric acid (10 mL) was added dropwise a solution of sodium nitrite (248 mg, 3.6 mmol) in water (5 mL) at 5 ^o^ C. After the addition, the mixture was stirred at ambient temperature for 15 min before the resulting solution was divided into 20 2-dram vials. To each vial was added a mixture of 2-aroylacetonitrile (0.15 mmol) and sodium acetate (74 mg, 0.90 mmol) in dioxane (1 mL). The vials were then vortexed at ambient temperature for 4 hrs, when the reaction mixtures were diluted by water (1 mL) and ethyl acetate (1 mL). The upper organic layer was taken by a pipette and transferred through a filter cartridge containing magnesium sulfate into a culture tube. The filtrates were concentrated in GeneVac and the residues were purified on silica SPE.

**3-110-5**: ^1^H NMR (600 MHz, CDCl_3_) δ 9.51 (br s, 1H), 8.67 (s, 1H), 8.03 (d, 1H, *J*=7.8), 7.99-7.97 (m, 2H), 7.90 (d, 1H, *J*=7.8), 7.76 (s, 1H), 7.67-7.7.65 (m, 1H), 7.62-7.58 (m, 2H), 7.54 (d, 1H, *J*=7.2), 7.43 (br s, 1H).

**3-110-14**: ^1^H NMR (600 MHz, CDCl_3_) δ 8.04 (s, 1H), 7.96-7.92 (m, 2H), 7.45 (s, 1H), 7.67-7.45 (m, 5H).

**3-110-22**: ^1^H NMR (600 MHz, CDCl_3_) δ 8.30 (s, 1H), 7.80 (d, 1H, *J*=8.4), 7.75 (s, 1H), 7.67-7.63 (m, 2H), 7.60-7.53 (m, 3H), 7.39-7.36 (m, 1H).

Analog ID: **3-110-1**, ESI-MS (m/z): 348.1; **3-110-2**, 394.1; **3-110-3**, 352.0; **3-110-4**, 318.1; **3-110-5**, 368.1; **3-110-6**, 378.1; **3-110-8**, 336.0; **3-110-9**, 343.0; **3-110-11**, 363.0; **3-110-12**, 346.1; **3-110-14**, 352.0; **3-110-15**, 362.1; **3-110-16**, 394.0, 396.0; **3-110-17**, 354.0; **3-110-20**, 399.1; **3-110-21**, 453.1; **3-110-22**, 358.1; **3-110-24**, 324.0; **3-110-25**, 388.1; **3-110-26**, 422.0.
