# Supplementary figures and images for: Correction: Small-Molecule Inhibitors of Dengue-Virus Entry
Source: PLoS Pathog. 2019 Jan 31;15(1):e1007553. doi: 10.1371/journal.ppat.1007553 (PMC6355023; doi:10.1371/journal.ppat.1007553)

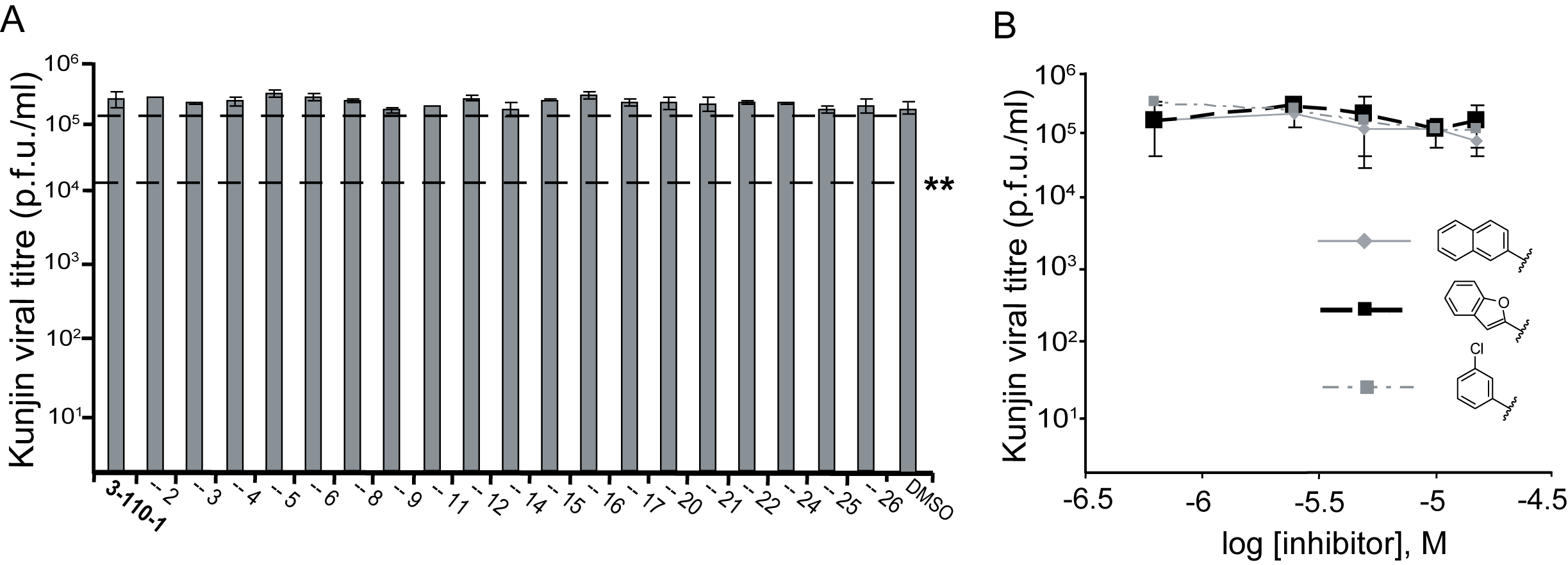

Supplement: S2 Fig — (TIF) [file ppat.1007553.s001.tif]

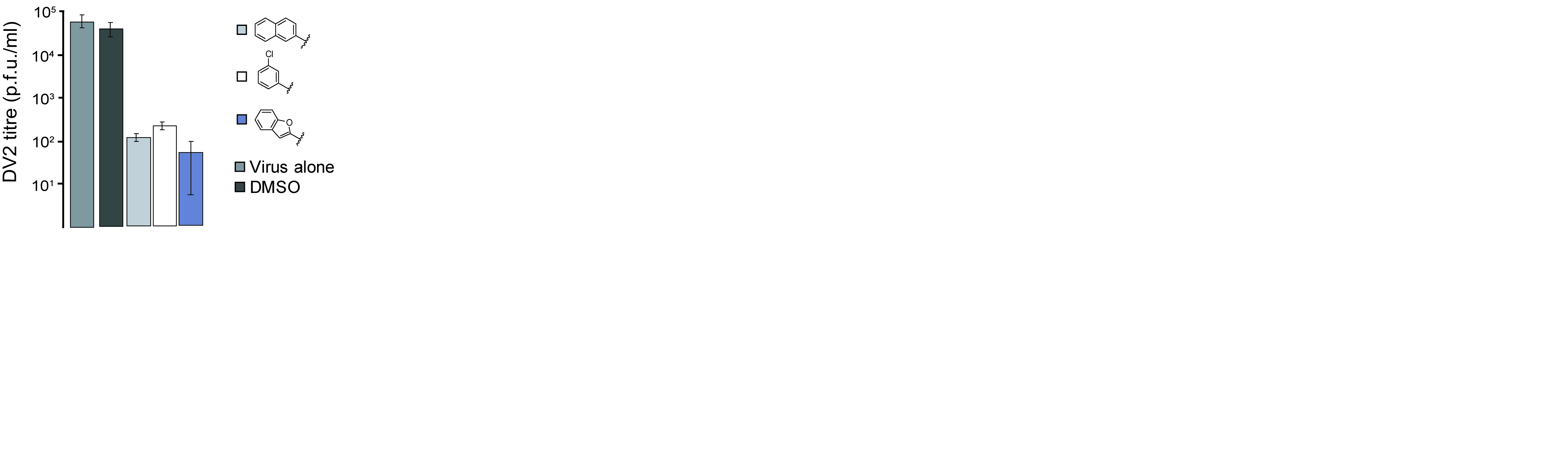

Supplement: S8 Fig — (TIF) [file ppat.1007553.s002.tif]

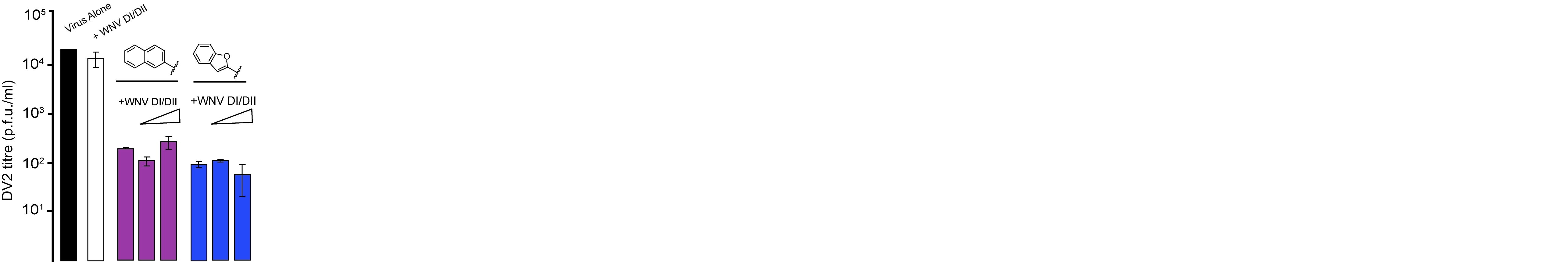

Supplement: S9 Fig — (TIF) [file ppat.1007553.s003.tif]
